# A Review on the Nomenclature and Taxonomy of the Old World Thread-Legged Bug Genus *Pleias* (Hemiptera: Reduviidae: Emesinae)

**DOI:** 10.3390/insects16010070

**Published:** 2025-01-12

**Authors:** Zhuo Chen, Hu Li, Wanzhi Cai

**Affiliations:** MOA Key Lab of Pest Monitoring and Green Management, Department of Entomology, College of Plant Protection, China Agricultural University, Yuanmingyuan West Road, Beijing 100193, China; insectchen625@126.com (Z.C.); tigerleecau@hotmail.com (H.L.)

**Keywords:** Heteroptera, new combination, new species, new synonym, Afrotropical Region, Oriental Region

## Abstract

The nomenclatural and taxonomic issues regarding the thread-legged bug genera *Pleias* Kirkaldy, 1901 and *Bagauda* Bergroth, 1903 are reviewed, and *Pleias* is concluded to be the valid name of the genus. A comprehensive review of *Pleias* is conducted, resulting in 18 new combinations and two new synonymies [*P. aelleni* (Villiers, 1970) **comb. n.**, *P. atypica* (Ghate, Boyane & Joshi, 2019) **comb. n.**, *P. avida* (Bergroth, 1903) **comb. n.**, *P. brunnea* (McAtee & Malloch, 1926) **comb. n.**, *P. cavernicola* (Paiva, 1919) **comb. n.**, *P. creppei* (Lhoste, 1939) **comb. n.** = *Bagauda gilletti* Miller, 1956 **syn. n.**, *P. ernstmayri* (Kulkarni & Ghate, 2016) **comb. n.**, *P. furcosa* (Ribes, 1987) **comb. n.**, *P. gigantea* (Lhoste, 1939) **comb. n.**, *P. lucifuga* (McAtee & Malloch, 1926) **comb. n.**, *P. monodi* (Villiers, 1972) **comb. n.**, *P. similis* (Wygodzinsky, 1966) **comb. n.**, *P. smithersi* (Wygodzinsky, 1966) **comb. n.**, *P. splendens* (Distant, 1906) **comb. n.**, *P. strinatii* (Villiers, 1970) **comb. n.**, *P. tenebricola* (Horváth, 1910) **comb. n.**, *P. wagneri* (Villiers, 1949) **comb. n.** = *B. eriksoni* Miller, 1954 **syn. n.**, and *P. zetteli* (Rédei, 2005) **comb. n.**]. The bibliographies, diagnosis, and known distribution records are presented for all described species, and three new species [*P. fashengi* **sp. n.** (from Yunnan, China), *P. serrata* **sp. n.** (from Sabah, Malaysia), and *P. trimaculata* **sp. n.** (from Sulawesi, Indonesia)] are described. The identification keys to the African and Asian species of *Pleias* are provided. The systematic relationships, distribution, and ecology of the genus are discussed.

## Simple Summary

The thread-legged bug genus *Bagauda* Bergroth, 1903 (Hemiptera: Reduviidae: Emesinae: Leistarchini) has native species in Africa and Asia, many of which are well known for their cave-living habits and have received attention in ecological, evolutionary, and taxonomic studies. The monotypic genus *Pleias* Kirkaldy, 1901 has recently been recognized as the senior synonym of *Bagauda*, yet the nomenclatural and taxonomic issues surrounding them have not been well resolved, resulting in the two genus-level names still being used as valid in current publications. This study presents a comprehensive review of the nomenclature and taxonomy of *Pleias* and its included species, providing a redescription of the genus and identification keys to the species, as well as bibliographies, diagnosis and known distributional records for each species, including the proposal of 18 new combinations, two new synonyms, and three new species.

## 1. Introduction

The thread-legged bug subfamily Emesinae (Hemiptera: Heteroptera: Reduviidae) is a morphologically and ecologically distinct group of terrestrial predatory insects, members of which typically possess elongate and slender bodies and appendages, as well as anteriorly extended raptorial forelegs, making them readily recognizable [1,2]. The Emesinae occurs in all zoogeographic regions, with high diversity in the tropics and subtropics and a large number of island endemic taxa [3,4]. They occupy a wide variety of microhabitats, including living plants, dead and drooping banna or fern fronds, tree trunks, and leaf litter [1,3,5]; many are adapted to live in caves or other dark environments [6,7,8]; and some are closely associated with web-building spiders, acting as predators, kleptoparasites, or both, and sometimes displaying interesting predatory behaviors such as tracking and luring [1,9,10,11]. The newest taxonomic scheme divided the Emesinae into six tribes (Collartidini, Emesini, Leistarchini, Oncerotrachelini, Saicini, and Visayanocorini), containing about 130 described genera and 1200 described species, ranking as one of the most species-rich subfamilies in the Reduviidae [2,12].

The emesine genus *Pleias* Kirkaldy, 1901 was originally described as monotypic for its type species, *P. ritsemae* Kirkaldy, 1901, from Sumatra [13]. The genus was considered a close relative of *Luteva* Dohrn, 1860 (currently a junior synonym of *Ploiaria* Scopoli, 1786) by Kirkaldy [13], but it was largely overlooked in subsequent works. The identity and taxonomic status of the genus have thus gradually become questionable, and it has either been speculated as a junior synonym of *Luteva* [14] or listed as a genus *incertae sedis* under the Leistarchini [1,15]. On the other hand, the genus *Bagauda* Bergroth, 1903, which initially contained only its type species *B. avidus* Bergroth, 1903 from India [16], has absorbed a number of African and Asian species in the nearly one hundred years since its description, and as of 2005 it has contained 18 species [1,15,17,18].

Based on the examination of the type material of *P. ritsemae*, Rédei [19] took the lead in clarifying the identities of the genus and the species, and he convinced the synonymy between *Pleias* and *Bagauda*. Even so, Rédei [19] did not propose relevant taxonomic changes but submitted an application to the International Commission on Zoological Nomenclature (ICZN), seeking to give the nomenclatural precedence of *Bagauda* over *Pleias* [20]. The Commission, however, decided to maintain the priority of *Pleias* after voting on the application [21]. After that, *Bagauda* was still used as a valid genus-level name in many taxonomic and faunistic works [8,22,23,24,25,26,27,28,29] as well as phylogenetic studies [2,12,30,31,32,33], while only Aukema et al. [34] applied *Pleias* and combined the species *B. zigzag* Rédei & Tsai, 2010 under this name. It could be seen that the ICZN [21] resolution has not been followed up but has largely been ignored by the scientific community. Since new species of the genus are still being discovered, and the genus is frequently included in ecological and systematic studies, a revision of its nomenclature and taxonomy is important.

To address the inconsistency in the use of the genus-level names, the nomenclatural and taxonomic issues surrounding *Pleias* and *Bagauda* are reviewed in the present study, and a comprehensive review of the genus is conducted. Eighteen new combinations and two new synonymies are proposed, with the bibliographies, diagnosis, and known distribution records of all described species summarized; three new species from Asia are described; the identification keys to the African and Asian species of the genus are provided; and the systematic relationships, distribution, and ecology of the genus are summarized and discussed.

## 2. Materials and Methods

### 2.1. Material Depository

Specimens examined or cited in the present study are deposited in the following institutions:
BMNHNatural History Museum, London, UKCAUEntomological Museum, China Agricultural University, Beijing, ChinaFMNHFinnish Museum of Natural History, Helsinki, FinlandMHNGMuséum d’Histoire Naturelle, Geneva, SwitzerlandMNHNMuséum National d’Histoire Naturelle, Paris, FranceNHMWNaturhistorisches Museum Wien, Vienna, AustriaNMNSNational Museum of Natural Science, Taichung, ChinaNZSINational Zoological Collection, Zoological Survey of India, Calcutta, IndiaRBINSInstitut Royal des Sciences Naturelles de Belgique, Brussels, BelgiumRMNHNaturalis Biodiversity Center, Leiden, The NetherlandsSDEISenckenberg Deutsches Entomologisches Institut, Müncheberg, GermanyUSNMUnited States National Museum of Natural History, Washington, DC, USAWZSIWestern Regional Station, Zoological Survey of India, Pune, IndiaZMUHZoologisches Institut und Zoologisches Museum, Universität von Hamburg, Hamburg, Germany

Label data of type specimens are copied verbatim in quotation marks (“ ”); lines on the same label are separated by a backslash (\); different labels are separated by a semicolon (;); and comments on label data are provided in square brackets ([ ]); printed (pr.) or handwritten (hw.) texts are indicated.

### 2.2. Morphological Study

Specimens were identified based on morphological characters by comparing with type specimens and/or following the identification keys in Wygodzinsky [1], Villiers [6], and others.

External morphological characters were examined using a Nikon SMZ745 stereoscopic microscope. Male and female genitalia were soaked in a heated 10% KOH solution for approximately ten minutes to remove soft tissue, rinsed in distilled water, and dissected under a stereoscopic microscope. Dissected genitalia were placed in a plastic vial containing glycerol and, after examination, pinned under the corresponding specimen.

Photographs were taken using a Canon 7D Mark II digital camera with a Canon macro lens EF 100 mm f/2.8L IS USM and MP-E 65 mm f/2.8 1-5X for habitus and an Olympus BX51 microscope for dissected body parts. Figures were stacked with Helicon Focus v.5.3 and assembled using Adobe Photoshop 2020. The distribution map was downloaded from the online version of SimpleMappr [35] and modified in Adobe Photoshop 2020.

Morphological terminology mainly follows Wygodzinsky [1] and Standring et al. [2]. Measurements were obtained using a calibrated micrometer. Distribution data are given from specimens examined in this study and literature records; for data from literature records without specimen examination, the sources are given; new records are marked by an asterisk (*).

### 2.3. Nomenclature

This paper and the nomenclatural acts it contains have been registered in Zoobank (www.zoobank.org), the official register of the International Commission on Zoological Nomenclature. The LSID (Life Science Identifier) number of the publication is: urn:lsid:zoobank.org:pub:C24DB52B-63F3-4578-9B2D-AAE06B71F8FA

## 3. Results

### 3.1. Nomenclatural and Taxonomic Issues Surrounding Pleias and Bagauda

The first to point out the synonymy between *Pleias* and *Bagauda* was Rédei [19]. He examined the type material of *P. ritsemae*, the type species of *Pleias*, and found that *Pleias* shared the entire set of diagnostic character states for defining *Bagauda* by Wygodzinsky [1], except for the forewing venation; thus it is insufficient to distinguish them as different genera. Rédei [19] argued that simply treating *Bagauda* as a junior synonym of *Pleias* by the law of priority would threaten the stability of nomenclature, and in particular, it would result in the 18 species included in *Bagauda* at that time being transferred to *Pleias* as new combinations. However, as the case failed to satisfy the condition of Art. 23.9.1.1 of the Code [36], Rédei [20] submitted an application to the Commission with a view to granting nomenclatural precedence of *Bagauda* over *Pleias*. The application was recommended by Forero [37], and Rédei and Tsai [38] described a new species, *B. zigzag*, prior to the ruling of the Commission. Later, the Commission ruled on Rédei’s application [20], with the result that *Pleias* should be maintained in precedence to *Bagauda* whenever the two are considered to be synonyms [21].

In subsequent works, only Aukema et al. [34] followed the ICZN [21] resolution and used *Pleias* as the senior synonym of the genus, while in others, *Bagauda* was still used as a valid genus-level name, frequently appearing in taxonomic [8,23,24,25,26,27,28,29,39], faunal [22,40], ecological [11,41], and phylogenetic [2,12,30,31,32,33] studies. Kulkarni and Ghate [26] briefly referred to the nomenclatural issue around *Pleias* and *Bagauda*, but they did not mention the ICZN [21] resolution and still described their new species under *Bagauda*. These conditions show that the ICZN [21] resolution is not well considered and implemented.

Although *Pleias* was rarely cited after publication, and *Bagauda* was undoubtedly in prevailing usage, the reason for this situation was rooted in the dubious taxonomic status of *Pleias* for some authors [1,14,15]. The conservation of *Bagauda* would request the Commission to use their plenary power, while this name has been referenced about 73 times in the last 150 years, and its child taxa have also been relatively less cited (see bibliographies under each species). Therefore, after the identity of *Pleias* has been clarified, sinking *Bagauda* as a junior synonym following the law of priority and proposing corresponding taxonomic changes would not cause a great upheaval in nomenclatural stability. Similar cases have been reported in other genus-level taxa of Reduviidae recently [42,43,44]. On the other hand, the Commission is the final authority in zoological nomenclature, and its resolutions on specific issues should be carefully upheld and applied by the scientific community.

In conclusion, we follow ICZN [21] and Aukema et al. [34] by treating *Pleias* as the valid genus name, with *Bagauda* as its junior synonym. This treatment results in 18 new combinations. Furthermore, after reviewing all described species of the genus, two new species-level synonyms are proposed.

### 3.2. Taxonomic Review of Pleias

**Genus ***Pleias*** Kirkaldy, 1901**

(Figure 1, Figure 2, Figure 3, Figure 4, Figure 5, Figure 6, Figure 7, Figure 8, Figure 9, Figure 10, Figure 11, Figure 12, Figure 13, Figure 14, Figure 15, Figure 16, Figure 17 and Figure 18)

*Pleias* Kirkaldy 1901: 56 [13] (original description); Bergroth (1906: 311) [14] (discussion); Wygodzinsky (1966: 219) [1] (record); Maldonado (1990: 119) [15] (catalogue); Rédei (2007: 60, 62) [19] (listed, discussion); Rédei (2008: 93) [20] (nomenclature); ICZN (2010: 338) [21] (nomenclature); Aukema et al. [34] (2013: 106) (catalogue, Palaearctic). **Type species** by monotypy: *Pleias ritsemae* Kirkaldy, 1901.

*Bagauda* Bergroth 1903: 12 [16] (original description); Distant (1903: 207) [45] (in key, redescription, distribution, fauna of India, Sri Lanka and Myanmar); Distant (1910: 176) [46] (listed, fauna of India, Sri Lanka and Myanmar); Jeannel (1919: 150, 155) [47] (in key, listed, fauna of East Africa); McAtee and Malloch (1926: 118, 138) [48] (in key, record, key to selected species, fauna of Philippines and Malaysia); Lhoste (1939: 2) [49] (redescription, key to selected species, fauna of Africa); Villiers (1948: 444, 451) [50] (in key, redescription, distribution, Afrotropical); Villiers (1949: 308, 327) [51] (in key, redescription, distribution, key to selected species, Afrotropical); Wygodzinsky (1958: 115, 142) [52] (in key, key to selected species, fauna of South Africa); Dispons (1965: 100) [53] (key to selected species, Oriental); Wygodzinsky (1966: 89, 94, 95) [1] (in key, redescription, distribution, key); Dispons (1970: 229) [54] (in key); Villiers (1970: 324) [6] (key, Oriental); Maldonado (1990: 98) [15] (catalogue); Livingstone and Ravichandran (1991: 27) [55] (in key, fauna of southern India); Ambrose (2006: 2396) [56] (listed, fauna of India); Rédei (2008: 93) [20] (nomenclature); ICZN (2010: 338) [21] (nomenclature); Rédei and Tsai (2010: 16) [38] (in key, diagnosis, distribution); Kulkarni and Ghate (2016: 366) [26] (listed); Chandra et al. (2017: 121) [25] (diagnosis, key to selected species, fauna of India); Ghate et al. (2019: 592) [27] (listed); Mukherjee et al. (2020: 38) [28] (catalogue, fauna of India); Joshi et al. (2022: 362) [29] (listed, fauna of India); Ranasinghe et al. (2024: 155) [8] (listed, fauna of India and Sri Lanka). Type species by monotypy: *Bagauda avidus* Bergroth, 1903. Synonymized by Rédei (2007: 62) [19].

**Diagnosis.** Recognized within Leistarchini by the following combination of character states: head lacking ventrolateral spiniferous processes; transverse interocular sulcus slightly curved posteriorly, not surpassing posterior margin of eyes; posterior lobe of pronotum completely covering mesonotum; fore femur armed ventrally with two series of spine-like setae arising from small, wart-like tubercles, and accessory series composed of small, setigerous tubercles and small, peg-like denticles; anteroventral series beginning some distance from base of segment, not interrupted at base, posteroventral series beginning very close to base of segment; fore tarsus three-segmented, with tarsomere I more than two times as long as tarsomeres II and III combined. Macropterous and micropterous forms are known.

**Redescription.** Macropterous male and female. ***Vestiture.*** Body surface smooth, dull to moderately shining, covered with tiny, decumbent pubescence; portion of head anteriad to antennifers and ventral surfaces of head and thorax with short, suberect pubescence; flagellomeres of antenna with dense, short, decumbent pubescence; dorsal and ventral surfaces of labium with rows of tiny, erect setae; apical half of dorsal surface of fore tibia and basal portion of ventral surface of fore tarsomere I with short, suberect to erect, golden setae; mid and hind tarsi and apexes of mid and hind tibiae with short, decumbent to suberect, golden setae; ventral surface of abdomen with sparse, long, suberect to erect pubescence.

***Structure.*** Head elongate oval to fusiform, moderately convex dorsally, flattened ventrally; anteocular region longer than postocular, with small, shallow, median depression dorsally before transverse interocular sulcus; lateral margins of postocular region rounded, or constricted at middle in dorsal view; transverse interocular sulcus not surpassing posterior margin of eyes. Eye medium to large-sized, protruding laterally, sometimes reaching ventral surface of head in lateral view. Antenna gracile, located beyond middle of anteocular region; scape longer than pedicel; basiflagellomere longer, as long as, or slightly shorter than distiflagellomere. Labium slender, straight to slightly curved; visible segments I and II subequal in length; visible segment III as long as, or longer than, visible segments I and II combined, tapering to apex.

Pronotum elongate, clearly divided into anterior and posterior lobes; anterior lobe subcylindrical, slightly narrowed posteriorly; posterior lobe trapezoidal, completely covering mesonotum, sometimes with weak, median, longitudinal carina on disc, posterior margin widely concave at midpoint. Scutellum small, exposed. Prosternum with rounded posterior margin.

Foreleg slender to relatively stout; coxa cylindrical; trochanter simple, with short, spine-like seta arising from indistinct tubercle at middle and near apex of inner surface, respectively; femur armed ventrally with two series of spine-like setae arising from small, wart-like tubercles, and accessory series composed of small, setigerous tubercles and small, peg-like denticles; anteroventral series beginning some distance from base of segment, not interrupted at base; posteroventral series beginning very close to base of segment; accessory series irregular, beginning at same level of posteroventral series, forming several rows at extreme apex of segment; tibia 0.6 times as long as femur, armed ventrally with one row of deflexed, spine-like processes; tarsus strongly sclerotized, three-segmented, slightly to distinctly more than half as long as tibia, with tarsomere I more than two times as long as tarsomeres II and III combined; claws paired, subequal in size. Mid and hind legs slender; hind femur surpassing apex of abdomen; mid and hind tarsi short, slender, with tarsomere III nearly as long as tarsomeres I and II combined; mid and hind claws curved, simple in shape.

Forewing elongate, relatively broad, not reaching or clearly surpassing apex of abdomen, with venation as usual for Leistarchini and rounded apical margin. Hind wing with transverse thickening more or less developed; hamus sharply approaching Sc + R and conjoined; m-cu sometimes very short; R + M approaching apex of wing; Cu extending shortly beyond m-cu, free ending or connected with R + M to form small cell, sometimes with short spur-like branch emitting from apex of cell.

Abdomen elongate, slightly narrowed at base. Tergite VII with nearly truncated to widely rounded posterior margin. Segment VIII in male clearly exposed.

Male genitalia: pygophore large; posterior margin simple, sometimes with short, flattened elevation, or triangular, truncated, or spine-like median process. Paramere in various lengths, slender, curved. Phallus symmetrical to asymmetrical; articulatory apparatus shorter than phallosoma, with basal plate arms widely separated or fused in varying degrees; phallosoma membranous, or with variously shaped sclerotizations, struts fused in majority of length; endosoma usually with complex spiny processes inside, sometimes asymmetrically arranged.

Female genitalia: Tergite VIII transverse, subsemicircular or subrectangular; tergite IX trapezoidal, nearly as long as, or longer than tergite VIII; valvifer I broad; valvula I small, subtriangular; valvula II well developed, sometimes clearly exposed; gonoplac short to elongate, finger-like, apically rounded, sometimes far surpassing apices of tergite IX and valvifers I.

Micropterous males and females are similar to macropterous forms. Pronotum with posterior lobe slightly reduced. Forewing minute, padlike.

**Diversity and distribution.** Including the newly proposed taxonomic changes and the newly described species, *Pleias* currently contains 23 species, six from the Afrotropical Region and 17 from the Oriental Region (Figure 19 and Figure 20).

***Pleias aelleni*** **(Villiers, 1970), comb. n.**

(Figure 1A,B)

*Bagauda aelleni* Villiers 1970: 323, 325 [6] (original description); Maldonado (1990: 99) [15] (catalogue, distribution); Ranasinghe et al. (2024: 155) [8] (redescription, distribution, record, ecology, discussion, photo). Holotype (♂): Sri Lanka, Uva, Rawanaella Cave, MHNG.

*Bagauda avidus*: Distant (1903: 208) [45] (description, distribution, figure). Misidentification.

**Type material examined. Holotype** (♂): “Rawanaella” [pr.]; “Bagauda [hw.] \ aelleni [hw.] \ n. sp. [hw.] \ A Villiers det 19 [pr.] 70 [hw.]”; “HOLO [hw.] \ TYPE [pr.]” [red rectangle] (MHNG). **Paratype**: 1♀, “grotte de Rawanaella \ près Ella (Ceylan) \ P. Strinati et \ V Aellen, 16-I-1970” [hw.]; “Bagauda [hw.] \ aelleni [hw.] \ n. sp. [hw.] \ A Villiers det 19 [pr.] 70 [hw.] ”; “PARATYPE” [pr., red rectangle] (MNHN).

**Additional material examined.** SRI LANKA: 1♀, E.E. Green (BMNH).

**Figure 1:**
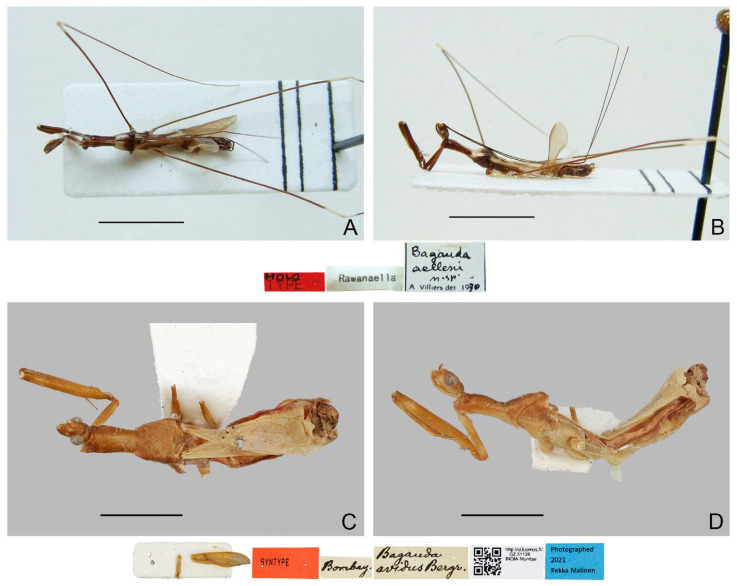
Type specimens of *Pleias* spp., habitus with labels. (**A**,**B**) *Pleias aelleni* (Villiers, 1970), **comb. n.**, male, holotype; (**C**,**D**) *Pleias avida* (Bergroth, 1903), **comb. n.**, female, lectotype (here designated). (**A**,**C**) Dorsal view; (**B**,**D**) lateral view. Scale bar of (**A**,**B**) = 5.0 mm; of (**C**,**D**) = 3.0 mm. ©MHNG (**A**,**B**) and ©FMNH (**C**,**D**).

**Diagnosis.** Recognized within the genus by the following combination of character states: relatively small-sized (9.0–10.5 mm); generally dark brown (Figure 1A,B); pronotum with contrasting dark and light color patterns as shown in Figure 1A; anterior lobe of pronotum 1.75 times as long as posterior lobe (Figure 1A,B); fore femur uniformly dark brown (Figure 1B); mid and hind femorotibial articulations whitish (Figure 1A,B); pygophore with simple posterior margin; paramere strongly curved.

**Distribution.** INDIA–Tamil Nadu: Sirumalai [8]. SRI LANKA–Eastern Prov.: Ampara [8]; North Central Prov.: Polonnaruwa [8]; Sabaragamuwa: Kegalle [8]; Uva: Rawanaella Cave; Western Prov.: Gampaha [8].

**Remarks.** In addition to the original description, Ranasinghe et al. [8] provided a redescription of this species, including the description and illustrations of the male genitalia. The color pattern on the pronotum shows intraspecific variation as shown in Ranasinghe et al. [8].

The Sri Lankan record of *B. avidus* by Distant [45] is based on a misidentification [1,29]. After examining the specimen illustrated by Distant [45], we confirmed that this specimen is a female of *P. aelleni* **comb. n.**

***Pleias atypica*** **(Ghate, Boyane & Joshi, 2019), comb. n.**

*Bagauda atypicus* Ghate, Boyane & Joshi 2019: 592 [27] (original description); Mukherjee et al. (2020: 38) [28] (catalogue, distribution); Joshi et al. (2022: 370) [29] (diagnosis, distribution, discussion). Holotype (♂): India, Maharashtra, Junnar Caves, WZSI.

**Diagnosis.** This is the only known micropterous species of *Pleias* that can be recognized within the genus by the following combination of character states: relatively small-sized (9.7–9.9 mm); generally blackish brown; anterior lobe of pronotum two times as long as posterior lobe; posterior lobe of pronotum with one pair of blunt, erect, humeral tubercles; mid and hind femorotibial articulations whitish; pygophore with short, triangular, apically acute median process.

**Distribution.** INDIA–Maharashtra: Junnar, Yeola Taluka [27].

***Pleias avida*** **(Bergroth, 1903), comb. n.**

(Figure 1C,D)

*Bagauda avidus* Bergroth 1903: 13 [16] (original description); Dispons [53] (1965: 101) (in key, distribution); Wygodzinsky (1966: 97) [1] (in key, distribution, discussion, type material, figure); Villiers (1970: 325) [6] (in key); Maldonado (1990: 99) [15] (catalogue, distribution); Ambrose (2003: 87) [57] (listed, ecology); Ambrose (2006: 2396) [56] (listed, distribution); Cao and Dang (2011: 228) [40] (record); Chandra et al. (2017: 121, 122) [25] (in key, redescription, distribution, record, photo); Mukherjee et al. (2020: 38) [28] (catalogue, distribution); Joshi et al. (2022: 362) [29] (redescription, distribution, record, type material, photo). Syntype (♀): India, Maharashtra, Mumbai, FMNH.

**Type material examined. Lectotype** (here designated) (♀): “SYNTYPE” [pr., red rectangle]; “Bombay” [hw.]; “Bagauda \ avidus Bergr.” [hw.]; “http://id.luomus.fi/\ GZ.51126 \ INDIA Mumbai” [pr.]; “Photographed \ 2021 \ Pekka Malinen” [pr., blue rectangle] (FMNH).

**Additional material examined.** MALDIVES: 1♂, Addu Atoll, Gan Island, 11.i.1959, W.W.A. Phillips (BMNH).

**Diagnosis.** Recognized within the genus by the following combination of character states: medium-sized (12.5–13.5 mm); generally brown, lacking conspicuous color patterns on pronotum, foreleg, and forewing (Figure 1C,D); anterior and posterior lobes of pronotum equal in length (Figure 1C,D); mid and hind femorotibial articulations whitish; forewing with cu-an1 meeting discal cell posterior to level of second r-m; pygophore with short, spine-like median process.

**Distribution.** INDIA–Karnataka: Sirsi [29]; Madhya Pradesh: Simariya [25]; Maharashtra: Lonavala [29], Mumbai; Tamil Nadu: Azhagar Kovil, Courtallam [56]. MALDIVES–Addu Atoll: Gan Island *. VIETNAM–Kon Tum: Chu Mom Ray [40].

**Remarks.** This is the type species of *Bagauda*. It was originally described based on an unspecified number of female specimens (syntypic) collected from “India orientalis (Bombay)” (= Mumbai, Maharashtra, India) [16]. One female specimen (Figure 1C,D), matching the original collection data and bearing E. Bergroth’s handwritten identification label and a red syntype label, was found in the collection of FMNH. This specimen is recognized as a syntype of *B. avidus* and designated herein as the lectotype of the species. This species was redescribed by Joshi et al. [29] based on both sexes, and they also provided illustrations of several morphological characters, including the male genitalia, of the species.

The record of this species from “Ceylon” (=Sri Lanka) by Distant [45] is based on a misidentification of *P. aelleni* **comb. n.** (see above). Therefore, the records from Sri Lanka by Distant [45] and subsequent authors [25,28,56] are herein excluded from the distribution of the species. The record of this species from Vietnam by Cao and Dang [40] is also in need of check. The species is newly recorded from Maldives.

***Pleias brunnea*** **(McAtee & Malloch, 1926), comb. n.**

*Bagauda brunneus* McAtee & Malloch 1926: 138, 139 [48] (original description); Dispons (1965: 101) [53] (in key, distribution); Wygodzinsky (1966: 95, 97) [1] (in key, listed, distribution, type material); Villiers (1970: 324) [6] (in key); Maldonado (1990: 99) [15] (catalogue, distribution). Holotype (♀): Philippines, Lanao de Norte, Kolambugan, USNM.

**Diagnosis.** Recognized within the genus by the following combination of character states: relatively small sized (9.5 mm); generally brown; fore femur uniformly brown; mid and hind femorotibial articulations brownish; forewing with cu-an1 meeting discal cell slightly posterior to level of second r-m, apical section of M as long as discal cell.

**Distribution.** PHILIPPINES–Lanao de Norte: Kolambugan [48].

***Pleias cavernicola*** **(Paiva, 1919), comb. n.**

*Bagauda cavernicola* Paiva 1919: 366 [58] (original description); Kemp and Chopra (1924: 13, 16) [59] (record, ecology); Kemp and China (1924: 94) [60] (ecology, discussion); Dispons (1965: 100) [53] (in key, distribution); Wygodzinsky (1966: 95, 97) [1] (in key, listed, distribution); Villiers (1970: 321, 324) [6] (listed, distribution, in key); Maldonado (1990: 99) [15] (catalogue, distribution); Ambrose (2006: 2396) [56] (listed, distribution); Chandra et al. (2017: 121) [25] (in key, redescription, distribution, photo); Harries et al. (2021: 125) [61] (record); Mukherjee et al. (2020: 38) [28] (catalogue, distribution); Joshi et al. (2022: 370) [29] (diagnosis, distribution, discussion). Holotype (sex unknown): India, Meghalaya, Siju Cave, NZSI.

**Material examined.** INDIA: 1♂2♀, Meghalaya, Garo Hills, Siju Cave, ii.1922, S. Kemp and B.N. Chopra (BMNH).

**Diagnosis.** Recognized within the genus by the following combination of character states: medium-sized (15.5–16.0 mm); generally dark brown; pronotum with contrasting dark and light color patterns; anterior lobe of pronotum 1.15 times as long as posterior lobe; fore femur with wide whitish annulus on apical third (except extreme apex); mid and hind femorotibial articulations whitish; forewing with apical section of M slightly shorter than discal cell.

**Distribution.** INDIA–Meghalaya: Siju Cave.

**Remarks.** Based on six specimens collected from “the Siju Cave, Garo Hills, Assam” (= Siju Cave, Meghalaya, India), Paiva [58] described *B. cavernicola* and designated the specimen “No. 8547/HI” as the holotype. The sex of the holotype was not mentioned in the original description, but it had been recorded that “antennae mutilated in type specimen”. A female with broken antennae was figured along with the original description, which might be the holotype of the species. However, our inquiries to NZSI were not given a response, thus the details of the holotype of this species were not yet known.

This species has not been rediscovered in recent investigations in its type locality [61]. The records of the species from Sri Lanka [25,28,56] lack precise locality and are probably erroneous.

***Pleias creppei*** **(Lhoste, 1939), comb. n.**

(Figure 2)

*Bagauda creppei* Lhoste 1939: 3, 4 [49] (original description); Villiers (1949: 329) [51] (in key, redescription, distribution, figure); Wygodzinsky (1966: 95, 97) [1] (in key, distribution, record, type material, figure); Maldonado (1990: 99) [15] (catalogue, distribution). Holotype (♀): Democratic Republic of the Congo, Sankuru, Tscheko Saka Cave, RBINS.

*Bagauda gilletti* Miller 1956: 53 [62] (original description); Gillett (1957: 193) [63] (ecology); Gillett (1958: 270) [64] (biological experiments); Wygodzinsky (1966: 95, 98) [1] (listed, distribution, type material); Maldonado (1990: 99) [15] (catalogue, distribution). Holotype (♂): Uganda, Central Region, Zika Forest, BMNH. **Syn. n.**

**Type material examined. ***Bagauda creppei*** Lhoste, 1939. Holotype** (♀): “Holotype” [pr., red rectangle]; “TYPE” [pr., red rectangle]; “TERRITOIRE [hw.] \ D [pr.] U [hw.] [pr.] \ SANKURU [hw.]”; “TSCHEKO-SAKA \ CONGO BELGE \ N. CREPPE” [pr.]; “Grotte C.B. 26 \ 23-5-1933 \ Z. 1.” [hw.]; “Bagauda [hw.] \ creppei [hw.] \ J. LHOSTE det [pr.] n. sp [hw.]”; “cf. Bull. Mus. [pr.] \ Hist. Nat. Belg. [pr.] \ XV, n°35, 1939 [hw.] \ p. 4–5 [hw.]” (RBINS).

***Bagauda gilletti *** **Miller, 1956. Holotype** (♂): “Holo- \ type” [pr., red-margined disc]; “Type” [pr., red-margined disc]; “♂” [pr.]; “ZIKA \ UGANDA \ JUNE 1954 \ J.D. GILLETT” [hw.]; “Bagauda [hw.] \ gilletti sp. n. [hw.] \ (holotype) [hw.] \ N.C.E. Miller det. 195 [pr.] 5 [hw.]”; “Brit. Mus. \ 1955-61.” [hw.]; “NHMUK 013587417” [pr.] (BMNH). **Paratype**: 1♀, “Para- \ type” [pr., yellow-margined disc]; “♀” [pr.]; “ZIKA \ UGANDA \ JUNE 1954 \ J.D. GILLETT” [hw.]; “Bagauda [hw.] \ gilletti sp. n. [hw.] \ (paratype) [hw.] \ N.C.E. Miller det. 195 [pr.] 5 [hw.]”; “Brit. Mus. \ 1955-61.” [hw.]; “NHMUK 013587418” [pr.] (BMNH).

**Figure 2:**
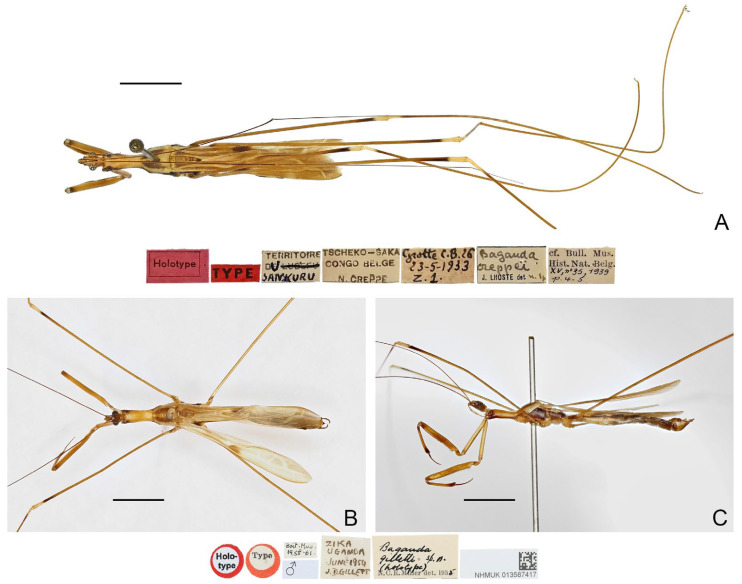
Type specimens of *Pleias* spp., habitus with labels. (**A**) *Pleias creppei* (Lhoste, 1939), **comb. n.**, female, holotype; (**B**,**C**) *Bagauda gilletti* Miller, 1956, male, holotype. (**A**,**B**) Dorsal view; (**C**) lateral view. Scale bars 5.0 mm.

**Diagnosis.** Recognized within the genus by the following combination of character states: large-sized (20.0–21.5 mm); generally yellowish brown (Figure 2A–C); anterior lobe of pronotum 1.15 times as long as posterior lobe (Figure 2A–C); posterior lobe of pronotum with one pair of lobe-like, dark-brown patches on disc (Figure 2A,B); mid and hind femorotibial articulations whitish (Figure 2A); forewing with distinct dark-brown suffusion at base of discal cell (Figure 2A,B); pygophore with long, spine-like median process.

**Distribution.** DEMOCRATIC REPUBLIC OF THE CONGO–Équateur: Eala [51]; Orientale: Kisangani [51]; Sankuru: Tscheko Saka Cave. UGANDA–Central Region: Buto Forest [1]; Zika Forest.

**Remarks.** Lhoste [49] described *B. creppei* based on a single female specimen (the holotype, Figure 2A) collected from “Congo Belge, Territoire du Sankuru, Tscheko-Saka” (=Tscheko Saka Cave, Sankuru, Democratic Republic of the Congo). The species was redescribed by Villiers [51], who also briefly illustrated the lateral view of the male genitalia. The holotype deposited in the collection of RBINS has been examined during this study.

Miller [62] described *B. gilletti* based on a male holotype (Figure 2B,C) and a female paratype collected from the Zika Forest of Uganda. He compared his new species with *B. creppei* and thought that *B. gilletti* differed from *B. creppei* in the coloration, the relative length of the anterior and posterior lobes of the pronotum, and the shape of the anterior angles of the posterior pronotal lobe [62]. However, Wygodzinsky [1] pointed out that this species could not be differentiated from *B. creppei*. The type material preserved in the collection of BMNH has been examined during this study.

A comparison between the type specimens of these two species suggests that they are conspecific, as no reliable differences in the coloration and the proportion of the anterior and posterior pronotal lobes could be found. Miller [62] mentioned that the anterior angles of the posterior pronotal lobe are more prominent in *B. creppei* than in *B. gilletti*, but such an interpretation might derive from the illustration of *B. creppei* by Lhoste [49], which does not match the condition of the holotype of the species. Since these two species could not be distinguished from each other based on morphological character states, the following new subjective synonymy is proposed: *Pleias creppei* (Lhoste, 1939), **comb. n.** = *Bagauda gilletti* Miller, 1956, **syn. n.**

***Pleias ernstmayri*** **(Kulkarni & Ghate, 2016), comb. n.**

*Bagauda ernstmayri* Kulkarni & Ghate 2016: 366 [26] (original description); Mukherjee et al. (2020: 39) [28] (catalogue, distribution); Joshi et al. (2022: 370) [29] (diagnosis, distribution, discussion). Holotype (♂): India, Maharashtra, Satara, WZSI.

**Diagnosis.** Recognized within the genus by the following combination of character states: medium-sized (12.8–13.3 mm); generally blackish brown; anterior lobe of pronotum 1.25 times as long as posterior lobe, basal half with wide, whitish-yellow annulus; fore femur with wide, whitish annulus subapically; fore tibia with narrow, whitish annulus subbasally; mid and hind femorotibial articulations whitish; forewing with distinct whitish patch basad of discal cell; pygophore with simple posterior margin.

**Distribution.** INDIA–Maharashtra: Satara [26].

**Remarks.** This species is very similar to *P. similis* (Wygodzinsky, 1966), **comb. n.** (see below), which can be separated, accordingly to Kulkarni and Ghate [26], by the differences in the color patterns on the pronotum and foreleg. The relationship between these two species deserves to be further studied based on more extended materials.

***Pleias furcosa*** **(Ribes, 1987), comb. n.**

(Figure 3A,B)

*Bagauda furcosus* Ribes 1987: 252 [17] (original description); Kerzhner (1993: 58) [65] (listed, distribution); Goula (2016: 166) [66] (listed); Serra et al. (2022: 12) [67] (type material, photo). Holotype (♂): Malaysia, Sarawak, Niah Cave, MHNG.

**Type material examined. Holotype** (♂): “Bagauda \ furcosus n. sp. ♂ \ RIBES 1986 \ HOLOTYPUS” [hw., red rectangle]; “Bagauda \ furcosus n. sp. ♂ \ J. Ribes 1986 \ HOLOTYPUS” [hw.]; “Traitement: \ KOH 10% \ Noir Chlorazol” [hw.]; “Tube \ A” [hw.] (MHNG).

**Additional material examined.** CHINA: 1♂, Yunnan, Mengla, Menglun, alt. 800 m, 21.iv.2007, Liangming Cao (CAU). MALAYSIA: 2♂1♀, Sarawak, Niah National Park, Niah Cave, v.1978, P. Chapman (BMNH).

**Figure 3:**
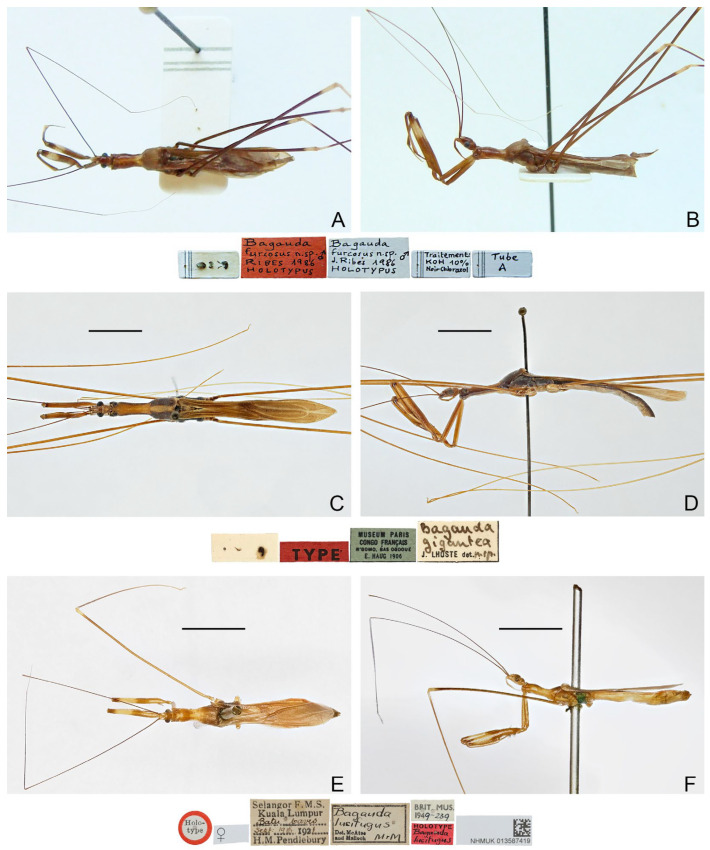
Type specimens of *Pleias* spp., habitus with labels. (**A**,**B**) *Pleias furcosa* (Ribes, 1987), **comb. n.**, male, holotype; (**C**,**D**) *Pleias gigantea* (Lhoste, 1939), **comb. n.**, male, holotype; (**E**,**F**) *Pleias lucifuga* (McAtee & Malloch, 1926), **comb. n.**, female, holotype. (**A**,**C**,**E**) Dorsal view; (**B**,**D**,**F**) lateral view. Scale bars 5.0 mm. ©MHNG (**A**,**B**).

**Diagnosis.** Recognized within the genus by the following combination of character states: medium-sized (14.0–17.0 mm); generally brown (Figure 3A,B); anterior lobe of pronotum 1.25 times as long as posterior lobe, with vague whitish-yellow stripe projecting forward from base (Figure 3A); fore femur with wide whitish annulus subapically (Figure 3A,B); mid and hind femorotibial articulations whitish (Figure 3A,B); forewing with cu-an1 meeting discal cell anterior to level of second r-m, apical section of M slightly shorter than discal cell; pygophore with one pair of small, robust, dorsal denticles; phallus with horn-like superoanterior process and one pair of apically rounded superolateral processes.

**Distribution.** CHINA–Yunnan: Mengla *. MALAYSIA–Sarawak: Niah Cave.

**Remarks.** This species was originally described based on specimens collected from the Niah Cave in Sarawak, Malaysia [17]. The holotype (Figure 3A,B) deposited in the collection of MHNG and several non-type specimens from its type locality in the collection of BMNH were examined during this study. A male specimen from Yunnan, China, was identified as the same species because it matched the former specimens in morphological characters, especially the remarkable male genital characters. The Chinese specimen differs in its smaller size (14 mm vs. more than 15 mm in Borneo specimens) and the incomplete light-colored subapical annulus on the fore femur.

***Pleias gigantea*** **(Lhoste, 1939), comb. n.**

(Figure 3C,D)

*Bagauda gigantea* Lhoste 1939: 3 [49] (original description); Villiers (1948: 452) [50] (redescription, distribution, figure); Villiers (1949: 329) [51] (redescription, distribution, figure). Holotype (♂): Gabon, Moyen-Ogooué, Ngomo, MNHN.

*Bagauda giganteus*: Wygodzinsky (1966: 95, 98) [1] (in key, diagnostic characters, distribution, type material); Villiers (1982: 31) [68] (record); Maldonado (1990: 99) [15] (catalogue, distribution).

**Type material examined. Holotype** (♂): “MUSEUM PARIS \ CONGO FRANÇAIS \ N’GOMO, BAS OGOOUE \ E. HAUG 1906” [pr.]; “Bagauda [hw.] \ gigantea [hw.] \ J. Lhoste det. [pr.] n. sp. [hw.]”; “TYPE” [pr., red rectangle] (MNHN).

**Additional material examined.** GHANA: 1♂, Western Region, Gold Coast, between Takoradi and Axim, 7.x.1944, P.A. Buxton (BMNH). NIGERIA: 1♀, Cross River, Oban, P.A. Talbot (BMNH).

**Diagnosis.** Recognized within the genus by the following combination of character states: large-sized (20.0 mm); generally yellowish brown (Figure 3C,D); anterior lobe of pronotum 1.5 times as long as posterior lobe (Figure 3C,D); posterior lobe of pronotum blackish brown except wide median band and posterior margin yellowish brown (Figure 3C,D); mid and hind femorotibial articulations whitish; forewing with indistinct dark-brown suffusion at base of discal cell; pygophore with long, spine-like median process.

**Distribution.** CENTRAL AFRICAN REPUBLIC–Lobaye: La Maboké [68]. GABON–Moyen-Ogooué: Ngomo. GHANA–Western Region: between Takoradi and Axim *. NIGERIA–Cross River: Oban *. REPUBLIC OF THE CONGO [68].

**Remarks.** This species is newly recorded from Ghana and Nigeria.

***Pleias lucifuga*** **(McAtee & Malloch, 1926), comb. n.**

(Figure 3E,F)

*Bagauda lucifugus* McAtee & Malloch 1926: 138 [48] (original description); Dispons (1965: 101) [53] (in key, distribution); Wygodzinsky (1966: 95, 98) [1] (in key, male genitalia, distribution, record, type material, figure); McClure et al. (1967: 419) [69] (record, ecology); Villiers (1970: 321, 324) [6] (listed, distribution, in key); Ribes (1987: 255) [17] (figure); Maldonado (1990: 99) [15] (catalogue, distribution); Moseley et al. (2012: 86) [22] (listed). Holotype (♀): Malaysia, Kuala Lumpur, Batu Caves, BMNH.

**Type material examined. Holotype** (♀): “Holo- \ type” [pr., red-margined disc]; “♀” [pr.]; “Selangor F.M.S. [pr.] \ Kuala Lumpur [pr.] \ Batu Caves [hw.] \ Sept. 19th [hw.] 192 [pr.] 1 [hw.] \ H.M. Pendlebury [pr.]”; “BRIT. MUS. [pr.] \ 194 [pr.] 9-239 [hw.]”; “Bagauda [hw.] \ lucifugus [hw.] \ Det. McAtee [pr.] \ and Malloch [pr.] M & M [hw.]”; “HOLOTYPE [pr.] \ Bagauda [hw.] \ lucifugus [hw.]” [red rectangle]; “NHMUK 013587419” [pr.] (BMNH). **Paratypes**: 1♂, “Para- \ type” [pr., yellow-margined disc]; “♂” [pr.]; “Selangor F.M.S. [pr.] \ Kuala Lumpur [pr.] \ Sept. 15th [hw.] 192 [pr.] 1 [hw.] \ H.M. Pendlebury [pr.]”; “BRIT. MUS. [pr.] \ 194 [pr.] 9-239 [hw.]”; “ALLOTYPE [pr.] \ Bagauda [hw.] \ lucifugus [hw.]” [red rectangle]; “NHMUK 013587420” [pr.] (BMNH). 1♂, “Para- \ type” [pr., yellow-margined disc]; “♂” [pr.]; “Selangor F.M.S. [pr.] \ Kuala Lumpur [pr.] \ Batu Caves [hw.] \ Sept. 15th [hw.] 192 [pr.] 1 [hw.] \ H.M. Pendlebury [pr.]”; “BRIT. MUS. [pr.] \ 194 [pr.] 9-239 [hw.]”; “Reduviid. \ Bagauda \ nr avidus \ (Bergr)” [hw.]; “PARATYPE [pr.] \ Bagauda [hw.] \ lucifugus [hw.]” [blue rectangle]; “NHMUK 013587421” [pr.] (BMNH). 2♀, “Para- \ type” [pr., yellow-margined disc]; “♀” [pr.]; “Selangor F.M.S. [pr.] \ Kuala Lumpur [pr.] \ Batu Caves [hw.] \ May 6th [hw.] 192 [pr.] 3 [hw.] \ H.M. Pendlebury [pr.]”; “BRIT. MUS. [pr.] \ 194 [pr.] 9-239 [hw.]”; “PARATYPE [pr.] \ Bagauda [hw.] \ lucifugus [hw.]” [blue rectangle]; “NHMUK 013587422” and “NHMUK 013587423” [pr.] (BMNH).

**Diagnosis.** Recognized within the genus by the following combination of character states: medium-sized (14.0–15.0 mm); generally light brown (Figure 3E,F); anterior lobe of pronotum 1.15 times as long as posterior lobe, with anteriorly bisinuate, whitish-yellow patch on basal half (Figure 3E,F); fore femur with wide whitish annulus subapically (Figure 3E,F); mid and hind femorotibial articulations whitish; forewing with cu-an1 meeting discal cell anterior to level of second r-m, apical section of M less than half of length of discal cell (Figure 3E); phallus with short superoanterior process and one pair of apically pointed superolateral processes, endosoma asymmetrical.

**Distribution.** MALAYSIA–Kuala Lumpur: Batu Caves.

***Pleias monodi*** **(Villiers, 1972), comb. n.**

(Figure 4A,B)

*Bagauda monodi* Villiers 1972: 396 [70] (original description); Maldonado (1990: 99) [15] (catalogue, distribution). Holotype (♂): Ethiopia, Oromiya, Sof Omar, MNHN.

**Type material examined. Holotype** (♂): “gr. Sof Omar \ Ethiopie \ 11.II.71” [hw.]; “Grotte de Sof Omar/Bale; Ethiopie \ (centre grotte) Th. Monod. 8.2.71 \ 14933-Reduviide” [hw.]; “Museum Paris [pr.] \ Th. Monod [hw.]”; “Bagauda [hw.] \ monodi [hw.] \ n. sp. [hw.] \ A Villiers det 19 [pr.] 72 [hw.]”; “HOLO [hw.] \ TYPE [pr.]” [red rectangle] (MNHN).

**Figure 4:**
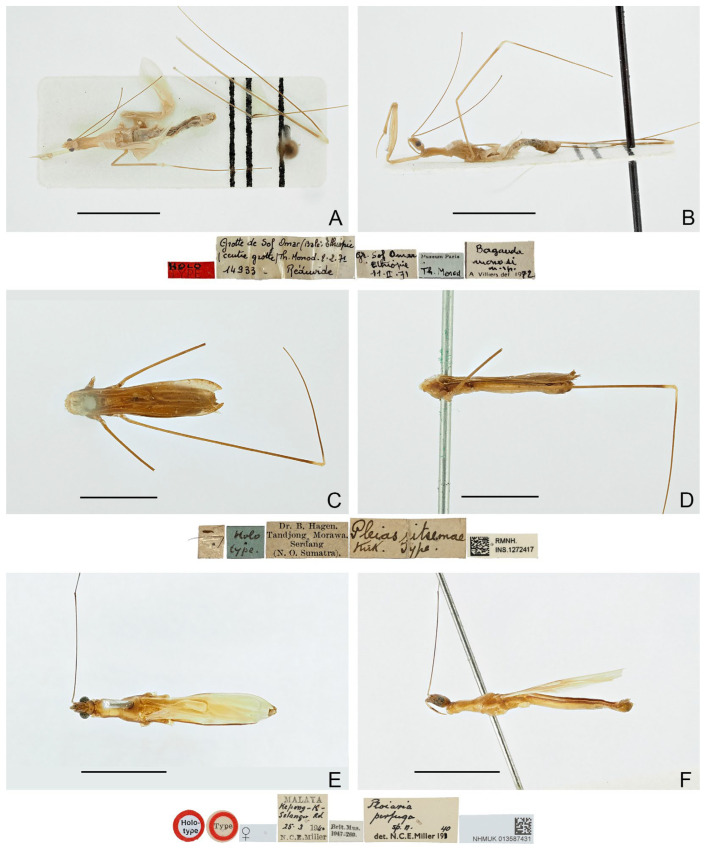
Type specimens of *Pleias* spp., habitus with labels. (**A**,**B**) *Pleias monodi* (Villiers, 1972), **comb. n.**, male, holotype; (**C**,**D**) *Pleias ritsemae* Kirkaldy, 1901, female, lectotype (here designated); (**E**,**F**) *Ploiaria perfuga* Miller, 1941, female, holotype. (**A**,**C**,**E**) Dorsal view; (**B**,**D**,**F**) lateral view. Scale bar of (**A**,**B**) = 5.0 mm; of (**C**–**F**) = 3.0 mm.

**Diagnosis.** Recognized within the genus by the following combination of character states: relatively small-sized (9.0 mm); generally light brown, lacking conspicuous color patterns on pronotum, foreleg, and forewing (Figure 4A,B); postocular region of head as long as eye (Figure 4B); labium with visible segment I not attaining anterior margin of eye (Figure 4B); anterior lobe of pronotum 1.15 times as long as posterior lobe (Figure 4A,B); mid and hind femorotibial articulations whitish (Figure 4A,B); pygophore with short, flat, apically truncated median process.

**Distribution.** ETHIOPIA–Oromiya: Sof Omar.

***Pleias ritsemae*** **Kirkaldy, 1901**

(Figure 4C–F)

*Pleias ritsemae* Kirkaldy 1901: 56 [13] (original description); Bergroth (1906: 311) [14] (discussion); Wygodzinsky (1966: 219) [1] (listed, distribution); Maldonado (1990: 119) [15] (catalogue, distribution); Rédei (2007: 60) [19] (diagnosis, redescription, distribution, type material, figure). Syntype (♀): Indonesia, North Sumatra, Tanjung Morawa, RMNH.

*Ploiaria perfuga* Miller 1941: 774, 776 [71] (original description); Wygodzinsky (1966: 219) [1] (listed, distribution, discussion, type material); Maldonado (1990: 120) [15] (catalogue, distribution). Holotype (♀): Malaysia, Selangor, between Kepong and Kuala Selangor, BMNH. Synonymized by Rédei (2007: 60) [19].

**Type material examined. ***Pleias ritsemae** *Kirkaldy, 1901. Lectotype** (here designated) (♀): “Dr. B. Hagen. \ Tandjong Morawa. \ Serdang \ (N.O. Sumatra.)” [pr.]; “Holo \ type” [hw.]; “Pleias ritsemae \ Kirk. Type.” [hw.]; “RMNH. \ INS.1272417” [pr.] (RMNH, Figure 4C,D).

***Ploiaria perfuga *** **Miller, 1941. Holotype** (♀): “Holo- \ type” [pr., red-margined disc]; “Type” [pr., red-margined disc]; “” [pr.]; “MALAYA [pr.] \ Kepong-K- [hw.] \ Selangor Rd [hw.] \ 25.3 [hw.] 19 [pr.] 40 [hw.] \ N.C.E. Miller [pr.]”; “Ploiaria [hw.] \ perfuga [hw.] \ sp. n. [hw.] \ det. N.C.E. Miller 19 [pr.] 40 [hw.]”; “Brit. Mus. \ 1947-269” [pr.]; “NHMUK 013587431” [pr.] (BMNH, Figure 4E,F).

**Diagnosis.** Recognized within the genus by the following combination of character states: small-sized (7.0–7.2 mm); generally yellowish brown, lacking conspicuous color patterns on pronotum, foreleg, and forewing (Figure 4C–F); anterior and posterior lobes of pronotum subequal in length (Figure 4E,F); forewing with M + Cu and cu-an1 forming characteristic arched vein at base of discal cell (Figure 4C,E).

**Distribution.** INDONESIA–North Sumatra: Tanjung Morawa. MALAYSIA–Selangor: between Kepong and Kuala Selangor.

**Remarks.** This is the type species of *Pleias*. It was described based on an unspecified number and sex of specimens (syntypic) collected from “Tandjong Morawa” (=Tanjung Morawa, North Sumatra, Indonesia) [13]. Based on a female specimen that apparently belongs to the type material, Rédei [19] provided a redescription and illustrations of the species and referred to it as “holotype”, possibly following the holotype label on the specimen. However, Kirkaldy [13] neither designated the holotype in the original description nor provided any indication on it, so this specimen is here treated as a syntype [36, Art 73.2], and the use of “holotype” by Rédei [19] did not constitute a valid lectotype designation [36, Art. 74.5]. In order to stabilize the nomenclature of the species, the specimen is designated herein as the lectotype of the species. The lectotype is severely damaged, with its anterior body parts missing.

***Pleias similis*** **(Wygodzinsky, 1966), comb. n.**

(Figure 5A–D)

*Bagauda similis* Wygodzinsky 1966: 97, 98 [1] (original description); Villiers (1970: 325) [6] (in key); Maldonado (1990: 99) [15] (catalogue, distribution); Ambrose (2003: 87) [57] (listed, ecology); Ambrose (2006: 2396) [56] (listed, distribution); Chandra et al. (2017: 121, 122) [25] (in key, redescription, distribution, photo); Mukherjee et al. (2020: 39) [28] (catalogue, distribution); Joshi et al. (2022: 371) [29] (diagnosis, distribution, discussion). Holotype (♂): India, West Bengal, Baigachhi, BMNH.

**Figure 5:**
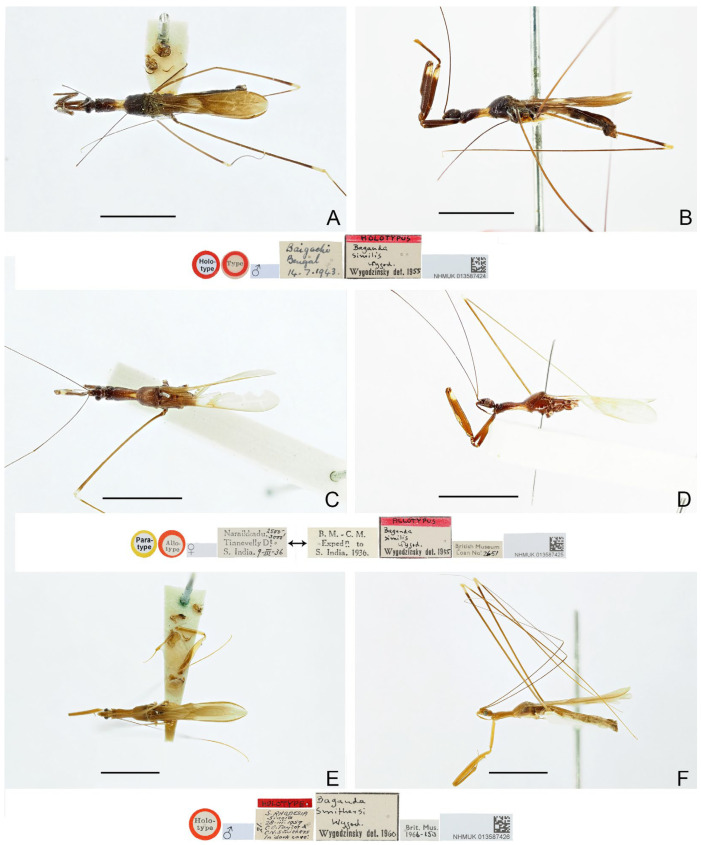
Type specimens of *Pleias* spp., habitus with labels. (**A**,**B**) *Pleias similis* (Wygodzinsky, 1966), **comb. n.**, male, holotype; (**C**,**D**) *Pleias similis* (Wygodzinsky, 1966), **comb. n.**, female, paratype; (**E**,**F**) *Pleias smithersi* (Wygodzinsky, 1966), **comb. n.**, male, holotype. (**A**,**C**,**E**) Dorsal view; (**B**,**D**,**F**) lateral view. Scale bars 5.0 mm.

**Type material examined. Holotype** (♂): “Holo- \ type” [pr., red-margined disc]; “Type” [pr., red-margined disc]; “♂” [pr.]; “Baigachi \ Bengal \ 14.7.1943.” [hw.]; “HOLOTYPUS [hw.] \ Bagauda [hw.] \ similis [hw.] \ Wygod. [hw.] \ Wygodzinsky det. 19 [pr.] 55 [hw.]”; “NHMUK 013587424” [pr.] (BMNH, Figure 5A,B). **Paratype**: 1♀, “Para- \ type” [pr., yellow-margined disc]; “Allo- \ type” [pr., red-margined disc]; “♀” [pr.]; “Naraikkadu, [pr.] 2500–3000′ [hw.] \ Tinnevelly Dt [pr.] \ S. India. [pr.] 9-III-36 [hw.]”, “B.M.–C.M. \ Expedn to \ S. India. 1936.” [pr., on reverse]; “ALLOTYPUS [hw.] \ Bagauda [hw.] \ similis [hw.] \ Wygod. [hw.] \ Wygodzinsky det. 19 [pr.] 55 [hw.]”; “British Museum [pr.] \ Loan No. [pr.] 2651 [hw.]”; “NHMUK 013587425” [pr.] (BMNH, Figure 5C,D).

**Diagnosis.** Recognized within the genus by the following combination of character states: medium-sized (12.0–12.5 mm); generally blackish brown (Figure 5A–D); anterior lobe of pronotum 1.25 times as long as posterior lobe, basal third with whitish-yellow patch with varying extension (occupying whole width in ♂, much smaller in ♀) (Figure 5A–D); fore femur with incomplete, wide, whitish annulus subapically (Figure 5B,D); mid and hind femorotibial articulations whitish (Figure 5A–D); forewing with distinct whitish patch basad of discal cell (Figure 5A,C); pygophore with simple posterior margin.

**Distribution.** INDIA–Kerala [28]; Tamil Nadu: Coimbatore [1], Naraikadu; West Bengal: Baigachhi.

**Remarks.** The size of the light-colored patch on the pronotum appears to be sexually dimorphic in this species, which occupies the whole width of the anterior lobe and connects the light-colored portion of the ventral surface in males (Figure 5A,B), whereas it is very small in females, being a middle spot (Figure 5C). This character state is the main basis to distinguish this species and *P. ernstmayri* **comb. n.**, but it is unclear whether variations exist among individuals.

***Pleias smithersi*** **(Wygodzinsky, 1966), comb. n.**

(Figure 5E,F)

*Bagauda smithersi* Wygodzinsky 1966: 97, 100 [1] (original description); Maldonado (1990: 99) [15] (catalogue, distribution). Holotype (♂): Zimbabwe, Mashonaland West, Chinhoyi, BMNH.

**Type material examined. Holotype** (♂): “Holo- \ type” [pr., red-margined disc]; “♂” [pr.]; “21. \ S. RHODESIA \ Sinoia \ 28.iii.1959 \ C.E. Taylor & \ C.N. Smithers \ In ‘dark cave’” [hw.]; “Bagauda [hw.] \ smithersi [hw.] \ Wygod. [hw.] \ Wygodzinsky det. 19 [pr.] 60 [hw.]”; “HOLOTYPE” [hw., red rectangle]; “Brit. Mus. [pr.] \ 196 [pr.] 6-153 [hw.]”; “NHMUK 013587426” [pr.] (BMNH, Figure 5E,F). **Paratypes**: 1♀, “Para- \ type” [pr., yellow-margined disc]; “Allo- \ type” [pr., red-margined disc]; “♀” [pr.]; “21 \ S. RHODESIA. \ Sinoia \ 28.iii.1959 \ C.E. Taylor & \ C.N. Smithers \ In ‘dark cave’” [hw.]; “Bagauda [hw.] \ smithersi [hw.] \ Wygod. [hw.] \ Wygodzinsky det. 19 [pr.] 60 [hw.]”; “ALLOTYPE” [hw., red rectangle]; “Brit. Mus. [pr.] \ 196 [pr.] 6-153 [hw.]”; “NHMUK 013587427” [pr.] (BMNH). 2♀, “Para- \ type” [pr., yellow-margined disc]; “♀” [pr.]; “21. \ S. RHODESIA. \ Sinoia \ 28.iii.1959 \ C.E. Taylor & \ C.N. Smithers \ In ‘dark cave’” [hw.]; “Bagauda [hw.] \ smithersi [hw.] \ Wygod. [hw.] \ Wygodzinsky det. 19 [pr.] 60 [hw.]”; “PARATYPE” [hw., red rectangle]; “Brit. Mus. [pr.] \ 196 [pr.] 6-153 [hw.]”; “NHMUK 013587428” and “NHMUK 013587429” [pr.] (BMNH).

**Diagnosis.** Recognized within the genus by the following combination of character states: medium-sized (12.0–13.0 mm); generally brown, lacking conspicuous color patterns on pronotum, foreleg, and forewing (Figure 5E,F); postocular region of head shorter than eye (Figure 5F); labium with visible segment I not attaining anterior margin of eye (Figure 5F); anterior lobe of pronotum 1.2 times as long as posterior lobe, with faint light-colored stripe along midline (Figure 5E,F); mid and hind femorotibial articulations whitish (Figure 5F); forewing with cu-an1 nearly as long as second r-m, section of Cu delimitating basal margin of discal cell slightly curved inwards of cell (Figure 5E); pygophore with narrow, triangular, apically rounded median process.

**Distribution.** ZIMBABWE–Mashonaland West: Chinhoyi.

***Pleias splendens*** **(Distant, 1906), comb. n.**

(Figure 6A–D)

*Bagauda splendens* Distant 1906: 364 [72] (original description); Distant (1910: 176) [46] (redescription, distribution); Kemp and China (1924: 94) [60] (ecology); Dispons (1965: 100) [53] (in key, distribution); Wygodzinsky (1966: 97, 101) [1] (in key, diagnostic characters, record, discussion, type material); Villiers (1970: 325) [6] (in key); Maldonado (1990: 99) [15] (catalogue, distribution); Chandra et al. (2015: 75) [24] (redescription, distribution, record, photo); Chandra et al. (2017: 121, 123) [25] (in key, redescription, distribution, record, photo); Mukherjee et al. (2020: 39) [28] (catalogue, distribution); Joshi et al. (2022: 371) [29] (diagnosis, distribution, discussion). Syntype (♂): Sri Lanka, Central Prov., Peradeniya, BMNH.

*Bagauda decorus* Breddin 1909: 301 [73] (original description). Syntype (♂): Sri Lanka, Southern Prov., Weligama, SDEI. Synonymized by Distant (1910: 176) [46].

**Figure 6:**
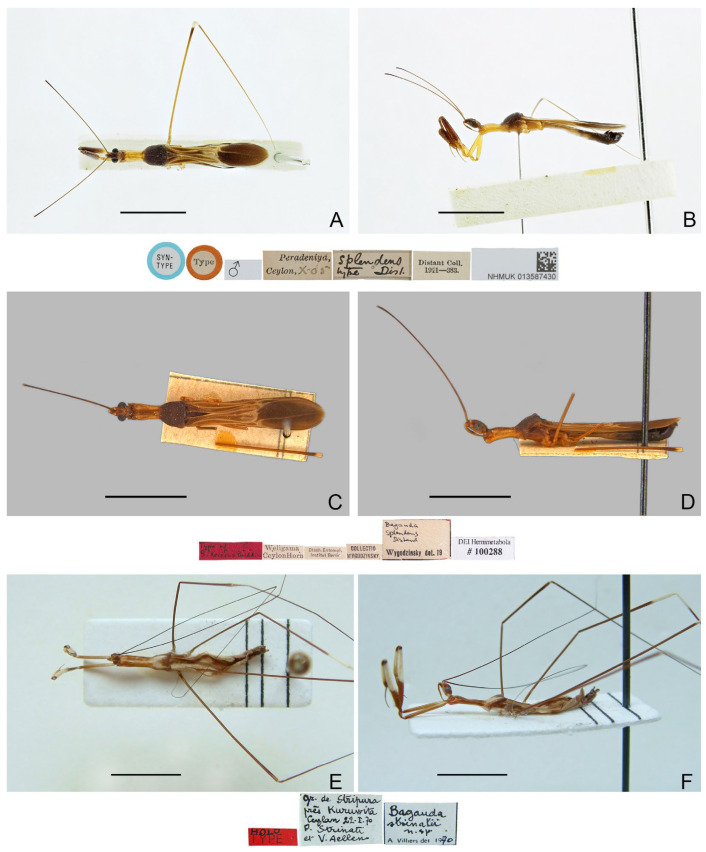
Type specimens of *Pleias* spp., habitus with labels. (**A**,**B**) *Pleias splendens* (Distant, 1906), **comb. n.**, male, lectotype (here designated); (**C**,**D**) *Bagauda decorus* Breddin, 1909, male, lectotype (here designated); (**E**,**F**) *Pleias strinatii* (Villiers, 1970), **comb. n.**, male, holotype. (**A**,**C**,**E**) Dorsal view; (**B**,**D**,**F**) lateral view. Scale bars 5.0 mm. ©SDEI (**C**,**D**) and ©MHNG (**E**,**F**).

**Type material examined. ***Bagauda splendens*** Distant, 1906. Lectotype** (here designated) (♂): “SYN- \ TYPE” [pr., blue-margined disc]; “Type” [pr., red-margined disc]; “♂” [pr.]; “Peradeniya, [pr.] \ Ceylon, [pr.] X-05 [hw.]”; “splendens \ Type Dist.” [hw.]; “Distant Coll. \ 1911-383.” [pr.]; “NHMUK 013587430” [pr.] (BMNH, Figure 6A,B).

***Bagauda decorus*** ** Breddin, 1909. Lectotype** (here designated) (♂): “Weligama \ CeylonHorn” [pr.]; “Dtsch. Entomol. \ Institut Berlin” [pr.]; “Type of ? \ B. decorus Breddin” [hw., red rectangle]; “COLLECTIO. \ WYGODZINSKY” [pr.]; “Bagauda [hw.] \ splendens [hw.] \ Distant [hw.] \ Wygodzinsky det. 19 [pr.]”; “DEI Hemimetabola \ # 100288” [pr.] (SDEI, Figure 6C,D).

**Additional material examined.** INDIA: 1♀, South India, T.V. Campbell (BMNH).

**Diagnosis.** Recognized within the genus by the following combination of character states: medium-sized (12.0–12.4 mm); generally orangish brown, with head, posterior lobe of pronotum, apical two-thirds of fore femur, fore tibia, forewing (except veins on corium), and apical half of abdomen dark brown to blackish (Figure 6A–D); anterior and posterior lobes of pronotum equal in length (Figure 6A–D); mid and hind femorotibial articulations whitish; forewing with cu-an1 meeting discal cell posterior to level of second r-m (Figure 6A,C); pygophore with simple posterior margin.

**Distribution.** INDIA–Madhya Pradesh: Chhindwara [24]. SRI LANKA–Central Prov.: Peradeniya; Southern Prov.: Weligama.

**Remarks.** This species is very distinct among its other Asian congeners due to the remarkable color patterns. It was originally described based on an unspecified number and sex of specimens (syntypic) collected from “Ceylon; Peradeniya” (=Peradeniya, Central Prov., Sri Lanka) [72]. A male specimen (Figure 6A,B), matching the original collection data and bearing a W.L. Distant’s handwritten identification label and a red-margined type label, was found in the collection of BMNH. This specimen is recognized as a syntype of *B. splendens* and designated herein as the lectotype of the species.

Breddin [73] described *B. decorus* based on an unspecified number of male specimens (syntypic) collected by W. Horn from Weligama in Sri Lanka. This species was synonymized with *B. splendens* shortly thereafter [46]. Wygodzinsky [1] located a specimen in the collection of SDEI, which he thought “is possibly the type of *decorus*”, but incorrectly recorded it as a female. During this study, a male specimen (Figure 6C,D) that matches the original collection data were found in the collection of SDEI. We consider it as a syntype of *B. decorus* and designate it herein as the lectotype of the species.

***Pleias strinatii*** **(Villiers, 1970), comb. n.**

(Figure 6E,F)

*Bagauda strinatii* Villiers 1970: 322, 325 [6] (original description); Kerzhner (1993: 52) [65] (nomenclature). Holotype (♂): Sri Lanka, Sabaragamuwa, Stripura Cave, MHNG.

*Bagauda strinatti*: Maldonado (1990: 99) [15] (catalogue, distribution). Incorrect subsequent spelling.

**Type material examined. Holotype** (♂): “gr. de Stripura \ près Kuruwita \ Ceylan 22.I.70 \ P. Strinati \ et V. Aellen” [hw.]; “Bagauda [hw.] \ strinatii [hw.] \ n. sp. [hw.] \ A Villiers det 19 [pr.] 70 [hw.]”; “HOLO [hw.] \ TYPE [pr.]” [red rectangle] (MHNG, Figure 6E,F).

**Diagnosis.** Recognized within the genus by the following combination of character states: medium-sized (11.5 mm); generally dark brown (Figure 6 E,F); pronotum with contrasting dark and light color patterns as shown in Figure 6E; anterior lobe of pronotum 1.85 times as long as posterior lobe (Figure 6E,F); apical half of fore femur whitish with apex blackish (Figure 6F); fore tibia whitish with base and apex blackish (Figure 6F); hind femorotibial articulation whitish (Figure 6E,F); pygophore with simple posterior margin; paramere strongly curved.

**Distribution.** SRI LANKA–Sabaragamuwa: Stripura Cave.

***Pleias tenebricola*** **(Horváth, 1910), comb. n.**

(Figure 7A,B)

*Bagauda tenebricola* Horváth 1910: 271 [74] (original description); Jeannel (1919: 155) [47] (record, ecology, figure); Lhoste (1939: 3) [49] (in key, diagnostic characters, figure); Villiers (1949: 329, 330) [51] (in key, redescription, distribution, figure); Miller (1953: 543, 563, 651) [75] (redescription, distribution, record); Kerzhner (1993: 52) [65] (nomenclature). Syntypes (1♂1♀, see remarks below): Tanzania, Tanga, Kulumuzi Cave, MNHN.

*Bagauda tenebricolus*: Wygodzinsky (1958: 113, 142, 143) [52] (in key, listed, distribution); Wygodzinsky (1966: 97, 102) [1] (in key, diagnostic characters, distribution, figure); Maldonado (1990: 99) [15] (catalogue, distribution).

**Figure 7:**
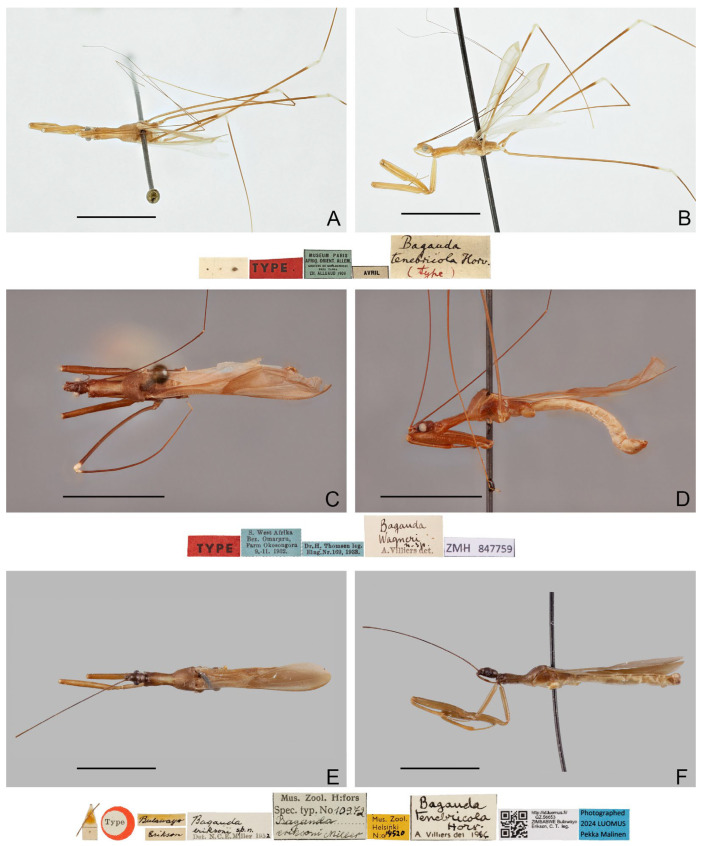
Type specimens of *Pleias* spp., habitus with labels. (**A**,**B**) *Pleias tenebricola* (Horváth, 1910), **comb. n.**, male, lectotype (here designated); (**C**,**D**) *Pleias wagneri* (Villiers, 1949), **comb. n.**, male, holotype; (**E**,**F**) *Bagauda eriksoni* Miller, 1954, female, holotype. (**A**,**C**,**E**) Dorsal view; (**B**,**D**,**F**) lateral view. Scale bars 5.0 mm. ©ZMUH (**C**,**D**) and ©FMNH (**E**,**F**).

**Type material examined. Lectotype** (here designated) (♂): “MUSEUM PARIS \ AFRIQ. ORIENT. ALLEM. \ GROTTES DE KOULOUMOUZI \ près TANGA \ CH. ALLUAUD 1909” [pr.]; “AVRIL” [pr.]; “Bagauda \ tenebricola Horv. \ (type)” [hw.]; “TYPE” [pr., red rectangle] (MNHN). **Paralectotype**: 1♀, “MUSEUM PARIS \ AFRIQ. ORIENT. ALLEM. \ GROTTES DE KOULOUMOUZI \ près TANGA \ CH. ALLUAUD 1909” [pr.]; “AVRIL” [pr.] (MNHN, Figure 7A,B).

**Additional material examined.** TANZANIA: 2♂, Tanga, Kulumuzi Cave, iv.1909, M.C. Alluaud (MNHN).

**Diagnosis.** Recognized within the genus by the following combination of character states: medium-sized (10.0–12.0 mm); generally brown, lacking conspicuous color patterns on pronotum, foreleg, and forewing (Figure 7A,B); postocular region of head as long as eye (Figure 7B); labium with visible segment I not attaining anterior margin of eye (Figure 7B); anterior lobe of pronotum 1.2 times as long as posterior lobe, with faint light-colored stripe along midline (Figure 7A,B); mid and hind femorotibial articulations whitish (Figure 7A,B); forewing with cu-an1 longer than second r-m, section of Cu delimitating basal margin of discal cell curved inwards to cell (Figure 7B); pygophore with wide, triangular, apically rounded median process.

**Distribution.** TANZANIA–Tanga: Kulumuzi Cave. ZIMBABWE–Manicaland: Odzi [75].

**Remarks.** This species was originally described based on “deux exemplaires trouvés dans la grotte de Kulumuzi, près de Tanga, par M. Ch. Alluaud” (=two specimens collected by M.C. Alluaud from the Kulumuzi Cave near Tanga) [74]. Four specimens that match the original collection data were found in the collection of MNHN, including three males and one female, and only one male (Figure 7A,B) bears a G. Horváth’s handwritten identification label and a red type label. Although only female was mentioned in the original description, the male that has G. Horváth’s handwritten identification label is clearly part of the type series. Here we consider the identification-label-bearing male and the only female of the four specimens as the type series of the species and designate the male as its lectotype. The type depository of the species was incorrectly given as the “Hungarian National Museum” in Wygodzinsky [1].

***Pleias wagneri*** **(Villiers, 1949), comb. n.**

(Figure 7C–F)

*Bagauda wagneri* Villiers 1949: 329, 331 [51] (original description); Wygodzinsky (1958: 113, 142, 143) [52] (in key, listed, distribution); Wygodzinsky (1966: 97, 102) [1] (in key, listed, distribution); Weidner (1972: 115) [76] (type material); Villiers (1973: 573) [77] (record); Maldonado (1990: 99) [15] (catalogue, distribution). Holotype (♂): Namibia, Erongo, Omaruru, ZMUH.

*Bagauda eriksoni* Miller 1954: 2 [78] (original description); Wygodzinsky (1958: 113, 142) [52] (in key, listed, distribution, discussion); Wygodzinsky (1966: 97, 98) [1] (in key, distribution, discussion, type material); Jansson and Coscarón (1989: 6) [79] (type material, discussion); Maldonado (1990: 99) [15] (catalogue, distribution). Holotype (♀): Zimbabwe, Bulawayo, FMNH. **Syn. n.**

**Type material examined. ***Bagauda wagneri** *Villiers, 1949. Holotype** (♂): “S. West Afrika \ Bez. Omaruru, \ Farm Okosongora \ 9.-11. 1932.” [pr.]; “Dr. H. Thomsen leg. \ Eing. Nr. 169, 1938.” [pr.]; “TYPE” [pr., red rectangle]; “Bagauda [hw.] \ Wagneri [hw.] \ n. sp. [hw.] \ A. Villiers det. [pr.]”; “ZMH 847759” [pr.] (ZMUH, Figure 7C,D).

***Bagauda eriksoni *** **Miller, 1954. Holotype** (♀): “Type” [pr., red-margined disc]; “Bulawayo” [hw.]; “Erikson” [hw.]; “Bagauda [hw.] \ eriksoni sp. n. [hw.] \ Det. N.C.E.Miller 195 [pr.] 2 [hw.]”; “Bagauda [hw.] \ tenebricola [hw.] \ Horv. [hw.] \ A Villiers det 19 [pr.] 64 [hw.]”; “Mus. Zool. [pr.] \ Helsinki [pr.] \ N:o [pr.] 14520 [hw.]” [yellow rectangle]; “Mus. Zool. H:fors [pr.] \ Spec. typ. No [pr.] 10972 [hw.] \ Bagauda [hw.] \ eriksoni Miller [hw.]”; “http://id.luomus.fi/\ GZ.56653 \ ZIMBABWE Bulawayo \ Erikson, C. T. leg.” [pr.]; “Photographed \ 2024 LUOMUS \ Pekka Malinen” [pr., blue rectangle] (FMNH, Figure 7E,F).

**Diagnosis.** Recognized within the genus by the following combination of character states: medium-sized (12.0–13.0 mm); generally brown, lacking conspicuous color patterns on pronotum, foreleg, and forewing (Figure 7C–F); postocular region of head longer than eye (Figure 7D,F); labium with visible segment I reaching anterior margin of eye, visible segment II surpassing posterior margin of eye (Figure 7F); anterior lobe of pronotum 1.2 times as long as posterior lobe, with faint light-colored stripe along midline (Figure 7C–F); mid and hind femorotibial articulations whitish (Figure 7C); forewing with section of Cu delimitating basal margin of discal cell nearly straight (Figure 7C,E); pygophore with narrow, triangular, apically rounded median process.

**Distribution.** NAMIBIA–Erongo: Omaruru; Otjozondjupa: Otavi [79]. ZIMBABWE–Bulawayo.

**Remarks.** *Bagauda wagneri* was originally described based on “Type: S.W. africain (Mus. Hambourg)”, without mention of its sex, but only male was described [51]. Later, Weidner [76] and Villiers [77] himself used the term “holotype” to refer to the specimen. It could be speculated that only one male specimen was used to describe the species, and the status of the specimen should be holotype.

*Bagauda eriksoni* was originally described based on a single female (the holotype) collected from Bulawayo, Zimbabwe [78]. It was thought to be closely allied to *B. wagneri* (see below) but differed in the shape of the anterior pronotal lobe [78]. Wygodzinsky [1] failed to distinguish the species with *B. wagneri* when constructing his identification key. Jansson and Coscarón [79], who examined the holotype of the species, mentioned that this specimen was identified as *B. tenebricola* by A. Villiers, but this putative synonymy has never been published.

The examination of the type specimens of *B. eriksoni* (Figure 7E,F) and *B. wagneri* (Figure 7C,D) shows identical morphological character states between the two species, and it was unclear what differences in the shape of the anterior pronotal lobe Miller [78] meant. Therefore, the following new subjective synonymy is proposed: *Pleias wagneri* (Villiers, 1949), **comb. n.** = *Bagauda eriksoni* Miller, 1954, **syn. n.** Although A. Villiers identified the holotype of *B. eriksoni* as conspecific with *B. tenebricola*, these two species can be separated by the different structures of the head, labium, and forewing venation, thus cannot be treated as synonyms.

***Pleias zetteli*** **(Rédei, 2005), comb. n.**

(Figure 8)

*Bagauda zetteli* Rédei 2005: 136 [18] (original description). Holotype (♂): Malaysia, Sabah, near Bilit, NHMW.

**Figure 8:**
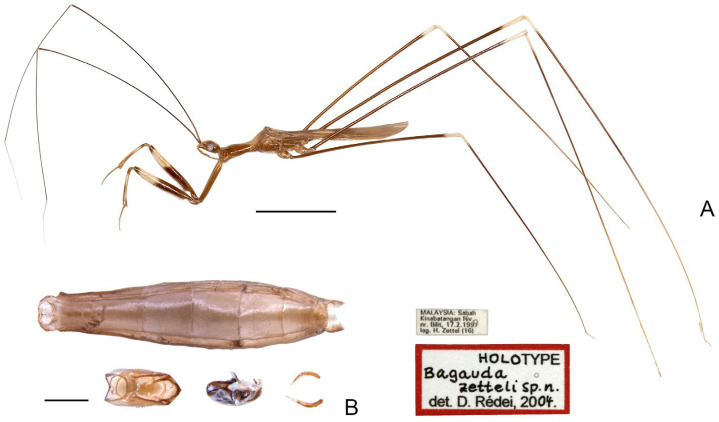
*Pleias zetteli* (Rédei, 2005), **comb. n.**, male, holotype. (**A**) habitus with labels, lateral view; (**B**) abdomen and male genitalia, dorsal view. Scale bar of (**A**) = 5.0 mm; of (**B**) = 1.0 mm. ©NHMW.

**Type material examined. Holotype** (♂): “MALAYSIA: Sabah \ Kinabatangan Riv. \ nr. Bilit, 17.2.1997 \ leg. H. Zettel (16)” [pr.]; “HOLO [hw.] TYPE [pr.] \ Bagauda [hw.] \ zetteli sp. n. [hw.] \ det. D. Rédei, 20 [pr.] 04 [hw.]. [pr.]” [red-margined rectangle] (NHMW, Figure 8).

**Additional material examined.** MALAYSIA: 2♂2♀, Sarawak, Gunung Mulu National Park, Deer Cave, iv.1978, P. Chapman, on cave wall near entrance (BMNH).

**Diagnosis.** Recognized within the genus by the following combination of character states: medium-sized (14.2–14.4 mm); generally dark brown (Figure 8A); pronotum with faint whitish-yellow patch occupying basal portion of anterior lobe and anterior half of posterior lobe; anterior lobe of pronotum 1.55–1.65 times as long as posterior lobe (Figure 8A); fore femur with wide whitish annulus subapically (Figure 8A); mid and hind femorotibial articulations whitish (Figure 8A); pygophore with posterior margin forming wide, flattened, projecting keel; phallus with one pair of apically narrowed superolateral processes.

**Distribution.** MALAYSIA–Sabah: Kinabatangan; Sarawak: Gunung Mulu National Park *.

***Pleias zigzag*** **(Rédei & Tsai, 2010)**

*Bagauda zigzag* Rédei & Tsai 2010: 17 [38] (original description). Holotype (♂): China, Taiwan, Kenting, NMNS.

*Pleias zigzag*: Aukema et al. (2013: 106) [34] (new combination, catalogue, distribution).

**Material examined.** CHINA: 2♀, Taiwan, Pingtung, Hengchun, Kenting National Park, 25.vii.2016, S.P. Wu and Y.T. Chung (CAU).

**Diagnosis.** Recognized within the genus by the following combination of character states: medium-sized (13.2–16.6 mm); generally dark brown; pronotum with distinct, extensive, whitish-yellow patch with deep bisinuate anterior and posterior margins; anterior lobe of pronotum 1.2–1.3 times as long as posterior lobe; fore femur with whitish annulus subapically; mid and hind femorotibial articulations whitish; pygophore with posterior margin bearing short, flattened, apically emarginated elevation; phallus with club-like dorsal projection on endosoma.

**Distribution.** CHINA–Taiwan: Pingtung.

### 3.3. Description of New Species

**Figure 9:**
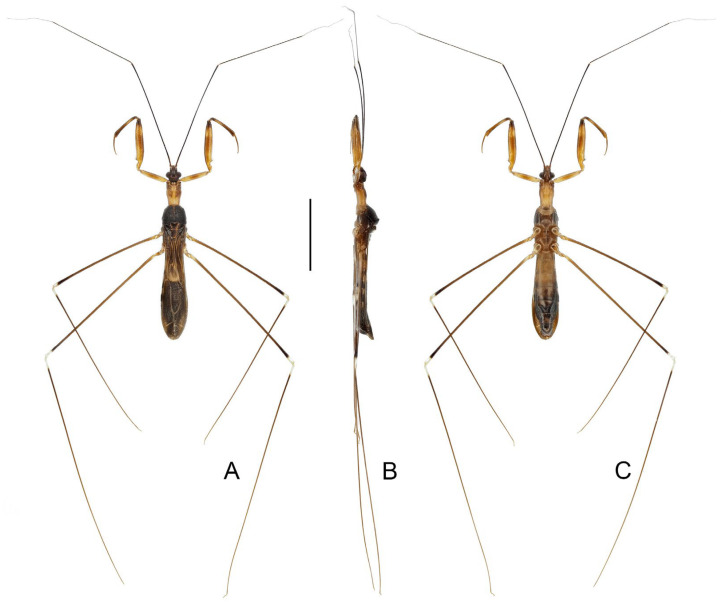
*Pleias fashengi*  **sp. n.**, male, paratype, habitus. (**A**) Dorsal view; (**B**) lateral view; (**C**) ventral view. Scale bar 5.0 mm.

**Figure 10:**
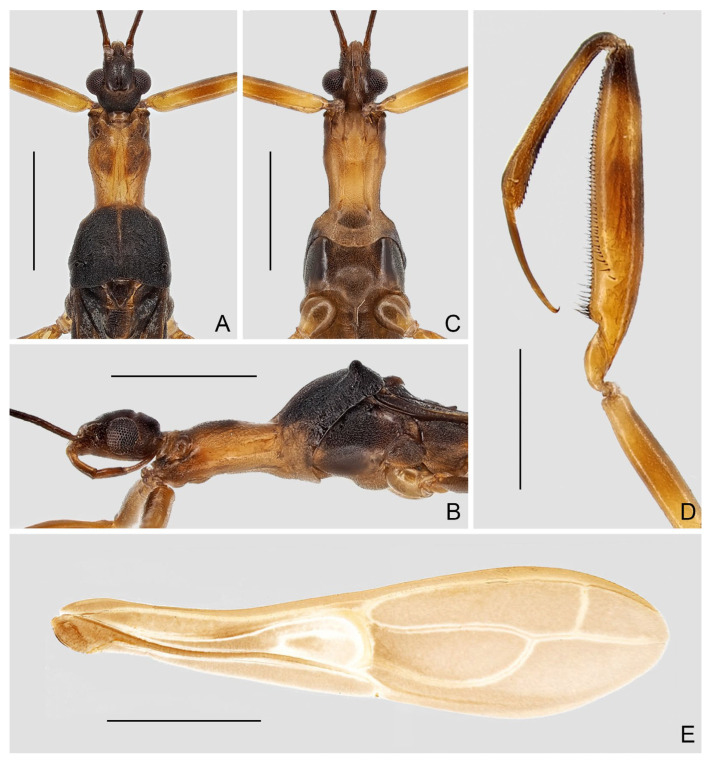
*Pleias fashengi*  **sp. n.**, male, paratype. (**A**–**C**) Anterior body parts; (**D**) foreleg; (**E**) forewing. (**A**,**E**) Dorsal view; (**B**) lateral view; (**C**,**D**) ventral view. Scale bar of (**A**–**C**,**E**) = 2.0 mm; (**D**) = 1.5 mm.

**Figure 11:**
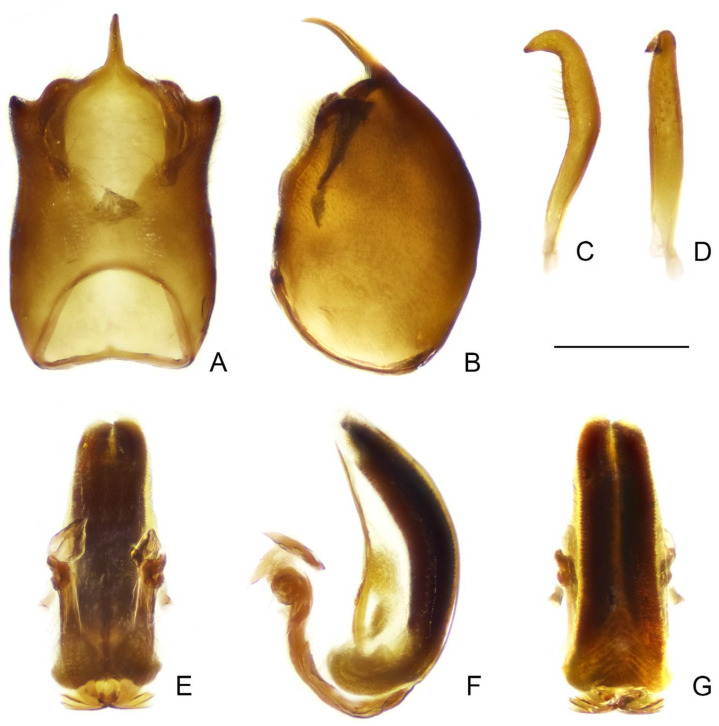
*Pleias fashengi*  **sp. n.**, male genitalia. (**A**,**B**) pygophore; (**C**,**D**) paramere; (**E**–**G**) phallus. (**A**,**E**) Dorsal view; (**B**,**F**) lateral view; (**G**) ventral view. Scale bar 1.0 mm.

**Figure 12:**
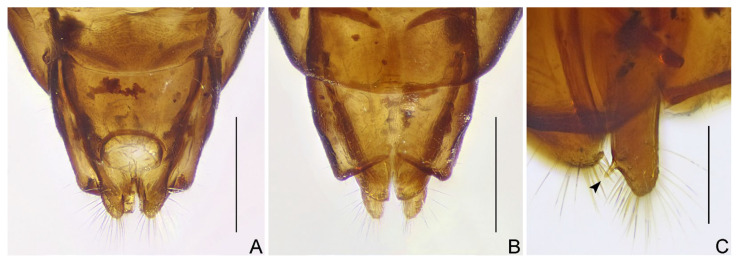
*Pleias fashengi*  **sp. n.**, female genitalia. (**A**) Dorsal view; (**B**) ventral view; (**C**) gonoplac, lateral view, arrow points to the finger-like process on ventral side. Scale bar of (**A**,**B**) = 1.0 mm; (**C**) = 0.5 mm.

***Pleias fashengi*** **sp. n.**

(Figure 9, Figure 10, Figure 11 and Figure 12)

*ZooBank LSID*: urn:lsid:zoobank.org:act:16771A53-99EE-44EB-BDBC-4F41BCA9945B

**Type material. Holotype** (♂): “Department of Plant Protection, Beijing Agricultural University [pr.] \ Yunnan, Jinghong [pr.] \ 1981-IV [pr.] -14 [hw.] \ Fasheng Li, 545 m [pr.]”; “♂” [pr.]; “HOLOTYPE [pr.] \ Pleias [hw.] \ fashengi sp. n. [hw.] \ Des. Chen, Li & Cai [pr.]” [red rectangle]; “CAU-RE-0000385 \ Ent. Mus. CAU. Beijing” [pr.] (CAU). **Paratypes**: 2♀, “CHINA Yunnan, Xishuangbanna, Jinghong, Jinuo \ Jimeng Rd., Yunfenggushan 1070m \ 2022-III-28 Zhaoyang Chen and Qinpeng Liu \ 22.058303E, 100.977634N \ Ent. Mus. CAU. Beijing” [pr.]; “♀” [pr.]; “PARATYPE” [pr., yellow rectangle]; “CAU-RE-0001600 \ Ent. Mus. CAU. Beijing” and “CAU-RE-0001601 \ Ent. Mus. CAU. Beijing” [pr.] (CAU); 1♂1♀, “CHINA Yunnan, Xishuangbanna, Menghai \ Man’ao 1250m \ E100.465653, N21.953057 \ 2022-VII-06 Zhaoyang Chen \ Ent. Mus. CAU. Beijing” [pr.]; “♂” or “♀” [pr.]; “PARATYPE” [pr., yellow rectangle]; “CAU-RE-0001602 \ Ent. Mus. CAU. Beijing” and “CAU-RE-0001603 \ Ent. Mus. CAU. Beijing” [pr.] (CAU); 2♂1♀, “CHINA Yunnan, Puer, Lancang \ Jingmaishan, Bulangyuni Inn \ 22.1807°N, 99.9901°E \ 2024-VIII-27 Haoyang Xiong \ Ent. Mus. CAU. Beijing” [pr.]; “♂” or “♀” [pr.]; “PARATYPE” [pr., yellow rectangle] (CAU).

**Diagnosis.** Medium-sized (11.5 mm) species which can be distinguished from its other congeners by the following character states: pronotum bicolorous with orangish-brown anterior lobe and black posterior lobe (Figure 10A,B); anterior and posterior lobes of pronotum equal in length (Figure 10A,B); humeral angles of pronotum prominently elevated (Figure 10B); fore femur orangish brown, with faint, wide, dark-colored annulus at middle and blackish-brown apex (Figure 10D); mid and hind femorotibial articulations whitish (Figure 9A,C); fore wing blackish brown with distinct light-colored markings, cu-an1 meeting discal cell posterior to level of second r-m (Figure 10E); abdominal connexivum bicolorous; pygophore with long, spine-like median process (Figure 11A,B).

**Description.** Macropterous male (Figure 9) and female. ***Coloration.*** Generally blackish brown (Figure 9). Head with ventral surface and portion anteriad to antennifers paler. Eye black. Antenna black. Labium brown to dark brown; basal portions of visible segments I and II paler. Pronotum conspicuously bicolorous; anterior lobe orangish brown, with anterior two-thirds faintly tinged with brown on both sides (Figure 10A,B); posterior lobe black, with anterior margin orangish brown (Figure 10A,B). Prosternum orangish brown (Figure 10C). Meso- and metapleura and sterna tinged with orange. Fore coxa orangish brown, with one faint, wide, dark-colored annulus at middle (Figure 10D); fore trochanter orangish brown; fore femur orangish brown, with one faint, wide, dark-colored annulus at middle, apically blackish brown (Figure 10D); fore tibia with one faint, subbasal, orangish brown annulus (Figure 10D); fore tarsus narrowly orangish brown basally (Figure 10D). Mid and hind coxae and trochanters orangish brown; mid and hind femora narrowly orangish brown basally, gradually darkened towards apex, blackish brown subapically, whitish apically (Figure 9A,C); mid and hind tibiae whitish basally, blackish brown subbasally, gradually lightened towards apex, light brown apically (Figure 9A,C); mid and hind tarsi light brown. Forewing blackish brown, with basal portion much darker; veins light brown to dark brown, with section of Cu delimiting basal margin of discal cell yellowish brown (Figure 10E); area basad of discal cell with whitish-yellow suffusion (Figure 10E). Hind wing light greyish-yellow with brown veins. Abdomen beneath whitish yellow on basal half, blackish brown on apical half, lacking distinct boundary between light and dark portions (Figure 9C); connexivum bicolorous, with each segment whitish yellow on basal half and blackish brown on apical half.

***Vestiture.*** As in redescription of the genus.

***Structure.*** Head (Figure 10A–C) 1.2 times as long as width across eyes, 2.1 times as broad across eyes as interocular space; anteocular region two times as long as postocular; lateral margins of postocular region rounded in dorsal view; interocular furrow bisinuate. Pronotum (Figure 10A–C) 1.6 times as long as width across humeral angles; anterior lobe as long as posterior lobe; humeral angles prominently elevated in lateral view, blunt tuberculated. Scutellum with round apical margin. Fore femur (Figure 10D) 1.7 times as long as fore coxa, 8.85 times as long as its maximum width; anteroventral series composed of about 54 spine-like setae arising from indistinct tubercles, length of seta gradually shortening towards apex of segment; posteroventral series composed of about 70 spine-like setae arising from indistinct tubercles; accessory series composed of small setigerous tubercles on basal fourth and small peg-like denticles on apical three-fourths of segment; fore tibia (Figure 10D) with about 34 deflexed spine-like processes ventrally; fore tarsus (Figure 10D) 0.6 times as long as fore tibia. Hind femur slightly shorter than body length (to apex of abdomen). Forewing (Figure 10E) with cu-an1 meeting discal cell posterior to level of second r-m; section of Cu delimiting basal margin of discal cell strongly curved inwards to cell; apical section of M slightly shorter than discal cell. Abdomen 2.6 times as long as its maximum width; segment VIII with anteromedial margin nearly straight and posteromedial margin strongly concave at midportion.

Male genitalia: Pygophore (Figure 11A,B) oblong, with an angled protrusion outside of each paramere insertion, and one pair of short rounded elevations on posterior margin; transverse bridge wide; median process long, spine-like, with acute apex. Paramere (Figure 11C,D) thin, curved at midpoint, strongly hooked at apex. Phallus (Figure 11E–G) elongated; articulatory apparatus short and slender, basal plate arms fused in basal half and widely separated in apical half, basal foramen small and oval, ponticulus basilaris short, dorsal connectives long; basal plate extension short; phallosoma elongated; endosoma symmetrical, with two rows of spiny processes inside.

Female genitalia: Tergite VIII broad, subsemicircular (Figure 12A); tergite IX nearly as long as tergite VIII (Figure 12A); valvifer I with rounded posterior margin (Figure 12B); valvula I with apex rounded (Figure 12B); valvula II slightly projecting posteriorly; gonoplac (Figure 12A–C) elongate, surpassing apices of valvifers I, with one finger-like, apically acute process nearly apex of ventral surface (Figure 12C).

**Measurements** [in mm, ♂ (n = 4)/♀ (n = 4)]. Length of body: to apex of fore wings 11.50–12.00/11.80–11.90, to apex of abdomen 11.00/12.00; length of head 1.20–1.25/1.30–1.40; length of anteocular region 0.50–0.55/0.50–0.55; length of postocular region 0.25–0.30/0.40; width across eyes 1.05–1.10/1.20; interocular space 0.50/0.70; length of antennal segments I–IV = 7.20–7.50/6.90–7.00, 5.70–6.20/5.20–5.50, 2.00–2.20/2.00–2.20, 1.30–1.40/1.50; length of visible labial segments I–III = 0.45–0.50/0.45, 0.40/0.40, 0.50/0.50; length of pronotum 2.40–2.60/2.40–2.60; length of anterior pronotal lobe 1.20–1.30/1.20–1.25; length of posterior pronotal lobe 1.20–1.30/1.20–1.35; width of anterior pronotal lobe 1.00–1.10/1.10; width of posterior pronotal lobe 1.50–1.75/1.60–1.65; median length of scutellum 0.40/0.40; basal width of scutellum 0.90/0.90; length of fore coxa, femur, tibia, tarsus = 1.80/1.70–1.80, 3.00–3.10/3.20, 1.90–2.00/1.90–2.00, 1.20–1.30/1.25–1.30; maximum width of fore femur 0.35–0.55/0.45–0.60; length of mid femur, tibia, tarsus = 7.40–7.70/7.30, 10.20–11.30/9.80–10.30, 0.30–0.40/0.30; length of hind femur, tibia, tarsus = 9.90–10.10/9.70–10.00, 15.00–16.50/14.00–14.80, 0.35–0.40/0.40; length of fore wing 7.50–8.00/7.90–8.20; length of abdomen 5.40–5.70/6.50–6.60; maximum width of abdomen 1.90–2.10/2.40.

**Etymology.** The specific epithet is dedicated to the Chinese entomologist Fasheng Li (1935–2021), to commemorate his contributions to Chinese insect taxonomy. He is also the collector of the holotype of the new species.

**Distribution.** CHINA–Yunnan: Jinghong, Lancang, Menghai.

**Comparative notes.** *Pleias fashengi* **sp. n.** is most similar to *P. avida* **comb. n.** and *P. splendens* **comb. n.** by sharing the relatively small body size, the equal length anterior and posterior lobes of the pronotum, the strongly inwardly curved Cu which delimitates the basal margin of the discal cell, and the cu-an1 meeting the discal cell posterior to the level of the second r-m.

*Pleias fashengi* **sp. n.** can be easily distinguished from *P. avida* **comb. n.** by its contrasting color patterns on the pronotum, foreleg, and forewing (vs. rather unicolored in *P. avida* **comb. n.**). The pygophore of the new species has a pair of angled protrusions and a pair of rounded elevations on its posterior margin, which are lacking in *P. avida* **comb. n.** In addition, the median process of the pygophore of the new species is longer than that in *P. avida* **comb. n.**, and the shape of their paramere is also different.

Both *P. fashengi* **sp. n.** and *P. splendens* **comb. n.** have the bicolorous pronotum and the contrasting markings on the forewing. The new species, however, markedly differs from the latter by the following character states: humeral angles of pronotum prominently elevated, forming small blunt protuberances (vs. slightly elevated, not forming protuberances in *P. splendens* **comb. n.**); fore femur mostly orangish brown, with faint, wide, dark-colored annulus at middle and blackish-brown apex (vs. mostly blackish brown, with basal fourth yellowish brown in *P. splendens* **comb. n.**); fore tibia blackish brown, with a faint orangish-brown annulus subbasally (vs. nearly uniformly blackish brown in *P. splendens* **comb. n.**); abdominal connexivum bicolorous (vs. unicolorous in *P. splendens* **comb. n.**); pygophore with long, spine-like median process (vs. lacking median process in *P. splendens* **comb. n.**).

The humeral angles of the pronotum in *Pleias* are usually slightly elevated but do not form distinct protuberances. The only exception is the recently described *P. atypica* **comb. n.**, which bears a pair of prominent humeral tubercles. The humeral protuberances of *P. fashengi* **sp. n.** are apparently weaker than those of *P. atypica* **comb. n.** The new species can be separated from *P. atypica* **comb. n.** by the larger body size, the completely different body color, and several male genital characters [27].

**Figure 13:**
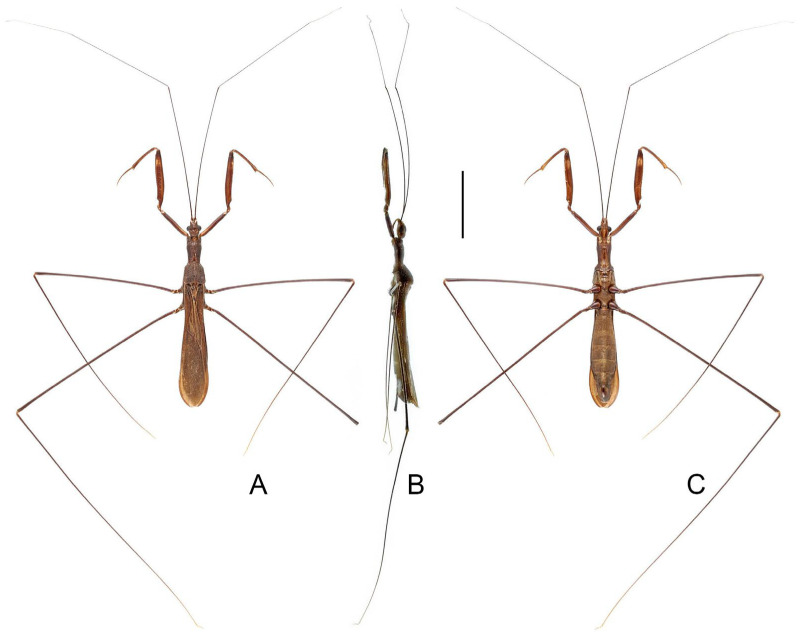
*Pleias serrata*  **sp. n.**, male, holotype, habitus. (**A**) Dorsal view; (**B**) lateral view; (**C**) ventral view. Scale bar 5.0 mm.

**Figure 14:**
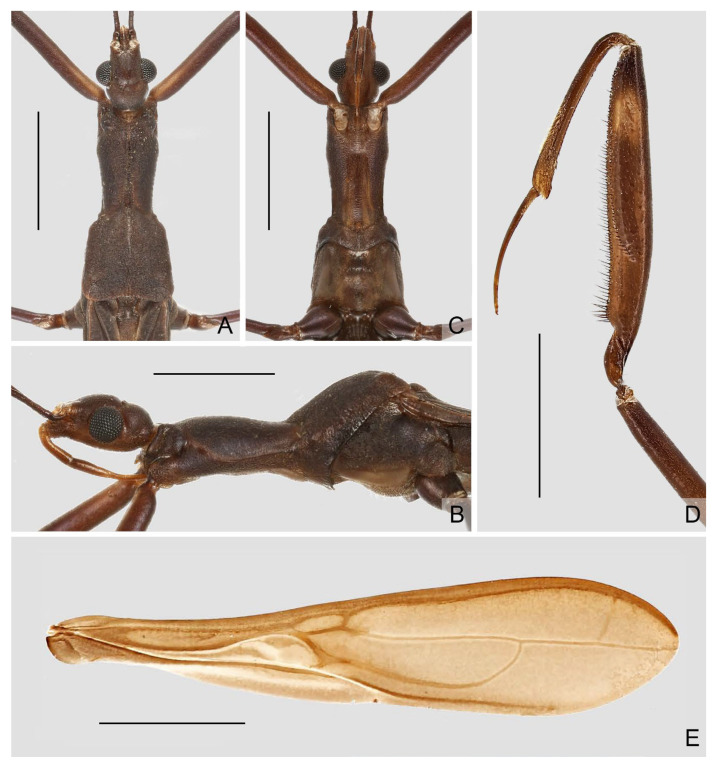
*Pleias serrata*  **sp. n.**, male, holotype. (**A**–**C**) Anterior body parts; (**D**) foreleg; (**E**) forewing. (**A**,**E**) Dorsal view; (**B**) lateral view; (**C**,**D**) ventral view. Scale bar of (**A**,**C**–**E**) = 2.0 mm; (**B**) = 1.5 mm.

**Figure 15:**
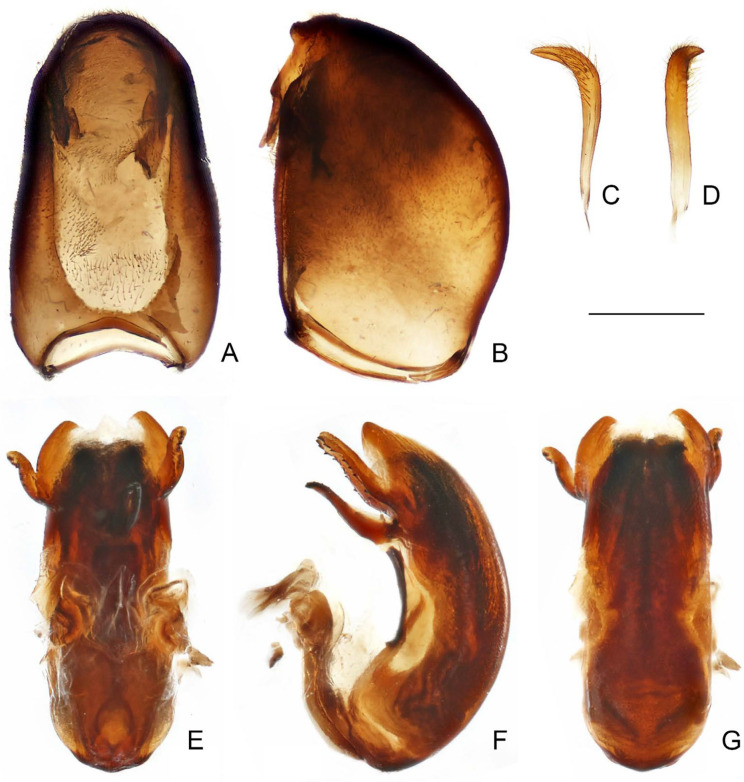
*Pleias serrata*  **sp. n.**, male genitalia. (**A**,**B**) pygophore; (**C**,**D**) paramere; (**E**–**G**) phallus. (**A**,**E**) Dorsal view; (**B**,**F**) lateral view; (**G**) ventral view. Scale bar 1.0 mm.

***Pleias serrata*** **sp. n.**

(Figure 13, Figure 14 and Figure 15)

*ZooBank LSID*: urn:lsid:zoobank.org:act:346A6DAB-1111-4AE8-A924-0D8982E450E9

**Type material. Holotype** (♂): “MALAYSIA Sabah, Mt. Trus \ Madi, Borneo Jungle Girl \ Camp 2017-V-1 Weiwei Zhang \ Ent. Mus. CAU. Beijing” [pr.]; “♂” [pr.]; “HOLOTYPE [pr.] \ Pleias [hw.] \ serrata sp. n. [hw.] \ Des. Chen, Li & Cai [pr.]” [red rectangle]; “CAU-RE-0001604 \ Ent. Mus. CAU. Beijing” [pr.] (CAU).

**Diagnosis.** Medium-sized (11.9 mm) species that can be distinguished from its other congeners by the following character states: body generally blackish brown (Figure 13); anterior lobe of pronotum slightly longer than posterior lobe, with distinct longitudinal furrow along midline (Figure 14A,B); fore femur with one incomplete, light-brown annulus subapically (Figure 14D); mid and hind femora unicolorous (Figure 13A,C); fore wing lacking contrasting light and dark color patterns, cu-an1 meeting discal cell anterior to level of second r-m (Figure 14E); pygophore with short, flattened, transverse elevation on posterior margin (Figure 15A,B); phallus with short, subrectangular dorsal phallothecal sclerite, one pair of horn-like superoanterior processes, and one pair of serrated, horn-like superolateral processes (Figure 15E–G).

**Description.** Macropterous male (Figure 13). ***Coloration.*** Generally blackish brown (Figure 13). Head with ventral surface and portion anteriad to antennifers paler. Eye black. Labium dark brown; visible segment III paler. Pronotum almost uniformly blackish brown (Figure 14A–C); visible part of metanotum slightly paler; ventral surface of thorax dark brown. Fore coxa with basal third brown and apical two-thirds dark brown, dorsum of basal third light brown; fore femur with one incomplete, light-brown annulus subapically (Figure 14D); fore tarsus dark brown (Figure 14D). Mid and hind tibiae dark brown, gradually lightened towards apex, yellowish brown apically; mid and hind tarsi yellowish brown. Forewing with area basad of discal cell and apical three-fourths of clavus faintly yellowish (Figure 14E); hind wing light greyish-yellow with brown veins. Abdomen uniformly dark brown, with connexivum darker.

***Vestiture.*** As in redescription of the genus.

***Structure.*** Head (Figure 14A–C) 1.4 times as long as width across eyes, two times as broad across eyes as interocular space; anteocular region 1.7 times as long as postocular; lateral margins of postocular region slightly constricted at middle in dorsal view; interocular furrow evenly curved backwards. Pronotum (Figure 14A–C) 1.7 times as long as width across humeral angles; anterior lobe slightly longer than posterior lobe, with shallow but distinct, median, longitudinal furrow containing many tiny particles; humeral angles rounded, weakly elevated in lateral view. Scutellum with apical margin nearly straight. Fore femur (Figure 14D) 1.5 times as long as fore coxa, 6.9 times as long as its maximum width; anteroventral series composed of about 48 spine-like setae arising from indistinct tubercles, length of seta gradually shortening towards apex of segment; posteroventral series composed of about 77 spine-like setae arising from indistinct tubercles; accessory series composed of small setigerous tubercles on basal two-thirds and small peg-like denticles on apical third of segment; fore tibia (Figure 14D) with about 36 deflexed spine-like processes ventrally; fore tarsus (Figure 14D) 0.7 times as long as fore tibia. Hind femur nearly equal to body length (to apex of abdomen). Forewing (Figure 14E) with cu-an1 meeting discal cell distinctly anterior to level of second r-m; apical section of M distinctly shorter than discal cell. Abdomen 3.4 times as long as its maximum width; segment VIII with anteromedial and posteromedial margins strongly concave.

Male genitalia: Pygophore (Figure 15A,B) elongate oval, with short, flattened, transverse elevation on posterior margin; transverse bridge narrow. Paramere (Figure 15C,D) thin, slightly curved, moderately acute at apex. Phallus (Figure 15E–G) robust, strongly sclerotized; articulatory apparatus thick, basal plate arms largely fused, basal foramen small and oval, ponticulus basilaris wide, dorsal connectives short; basal plate extension short; phallosoma with short, subrectangular dorsal phallothecal sclerite, one pair of long, parallel, band-like ventral sclerites narrowing apically, one pair of slender, horn-like superoanterior processes curved upwards, and one pair of robust, serrated, horn-like superolateral processes; endosoma symmetrical, with complex arrangements inside.

**Measurements** [in mm, ♂ (n = 1)]. Length of body: to apex of forewings 12.20, to apex of abdomen 11.90; length of head 1.40; length of anteocular region 0.60; length of postocular region 0.35; width across eyes 1.00; interocular space 0.50; length of antennal segments I–IV = 9.00, 7.10, ? (missing), ? (missing); length of visible labial segments I–III = 0.50, 0.50, 0.55; length of pronotum 2.70; length of anterior pronotal lobe 1.40; length of posterior pronotal lobe 1.30; width of anterior pronotal lobe 1.00; width of posterior pronotal lobe 1.60; median length of scutellum 0.30; basal width of scutellum 1.00; length of fore coxa, femur, tibia, tarsus = 2.50, 3.80, 2.20, 1.50; maximum width of fore femur 0.55; length of mid femur, tibia, tarsus = 9.10, 13.10, 0.50; length of hind femur, tibia, tarsus = 12.00, 18.80, 0.50; length of fore wing 8.00; length of abdomen 6.20; maximum width of abdomen 1.80.

**Etymology.** The specific epithet is derived from the Latin *serrata*, referring to the paired, serrated, horn-like superolateral processes on the phallus of the new species.

**Distribution.** MALAYSIA–Sabah: Mt. Trus Madi.

**Comparative notes.** Most species of *Pleias* present whitish mid and hind femorotibial articulations, while the unicolored mid and hind femora are quite rare within the genus, which is known only from *P. brunnea* **comb. n.** from the Philippines. The new species also shares the distinct median longitudinal furrow on the anterior pronotal lobe with the latter species. Despite the male of *P. brunnea* **comb. n.** remaining unknown, the new species is certainly not conspecific with this species due to the following character states: much larger body size (about 12 mm in *P. serrata* **sp. n.** vs. about 9.5 mm in *P. brunnea* **comb. n.**); fore femur with one incomplete, subapical, light-brown annulus (vs. fore femur unicolored in *P. brunnea* **comb. n.**); forewing with cu-an1 meeting discal cell distinctly anterior to level of second r-m (vs. slightly posterior to level of second r-m in *P. brunnea* **comb. n.**), and apical section of M much shorter than discal cell (vs. equal in length in *P. brunnea* **comb. n.**).

The two other species previously reported in Borneo, *P. furcosa* **comb. n.** and *P. zetteli* **comb. n.**, are large-sized species with body lengths over 14 mm, while *P. serrata* **sp. n.** is much smaller. These two species are also different from the new species in their apically whitish mid and hind femora and several male genital characters. In *P. furcosa* **comb. n.**, the pygophore bears one pair of sharp denticles on the dorsum (vs. such denticles are completely absent in *P. serrata* **sp. n.**); the parameres are evenly curved (vs. strongly curved in *P. serrata* **sp. n.**); the phallotheca has one pair of simply, apically rounded superolateral processes (vs. with curved, serrated, horn-like superolateral processes in *P. serrata* **sp. n.**). In *P. zetteli* **comb. n.**, the parameres are evenly curved, with a fine keel on the medial surface and a small subapical tubercle (vs. strongly curved, lacking a keel and tubercle in *P. serrata* **sp. n.**); the phallotheca has a narrow dorsal sclerotized plate and one pair of lobe-like superolateral processes that are narrowing and pointed apically (vs. the dorsal sclerotized plate is short and subrectangular, and the superolateral processes are horn-like and serrate, and there is one pair of horn-like superoanterior processes in *P. serrata* **sp. n.**).

**Figure 16:**
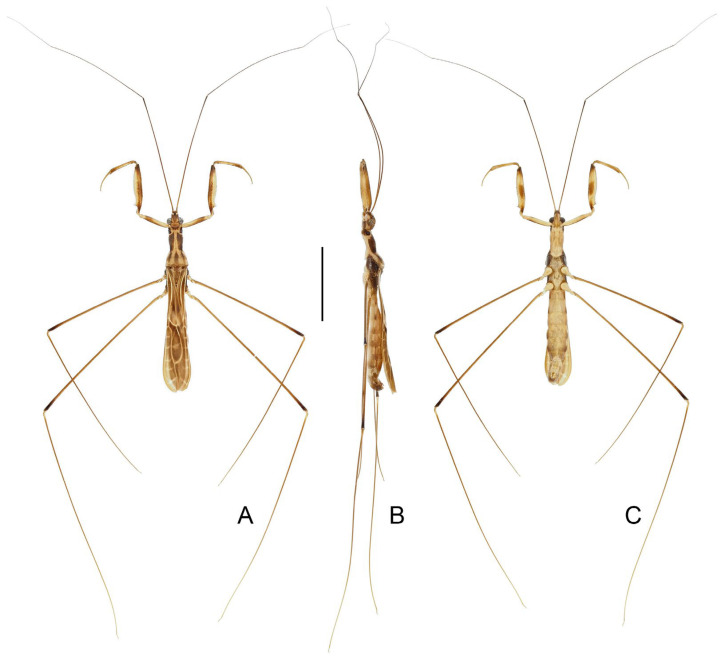
*Pleias trimaculata*  **sp. n.**, male, holotype, habitus. (**A**) Dorsal view; (**B**) lateral view; (**C**) ventral view. Scale bar 5.0 mm.

**Figure 17:**
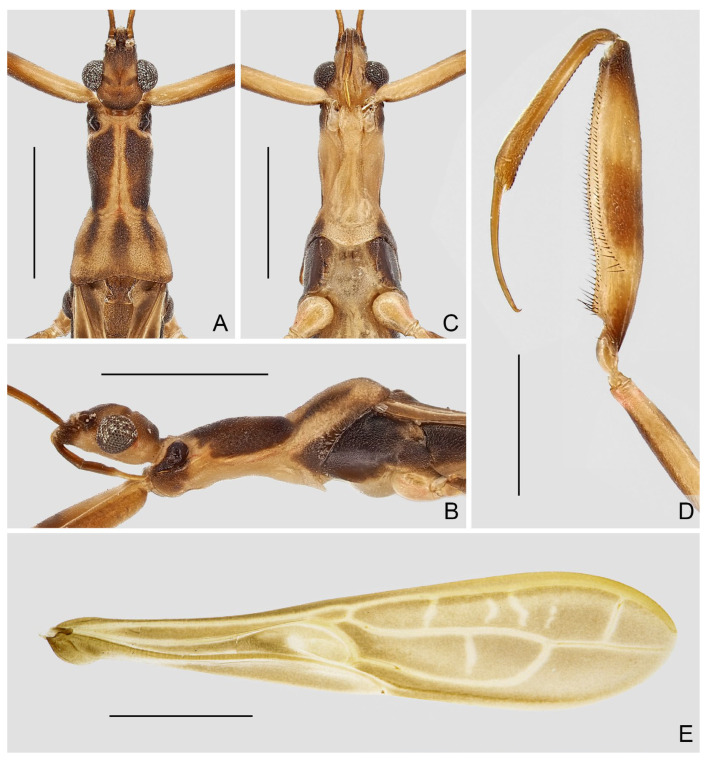
*Pleias trimaculata*  **sp. n.**, male, holotype. (**A**–**C**) Anterior body parts; (**D**) foreleg; (**E**) forewing. (**A**,**E**) Dorsal view; (**B**) lateral view; (**C**,**D**) ventral view. Scale bar of (**A**–**C**,**E**) = 2.0 mm; (**D**) = 1.5 mm.

**Figure 18:**
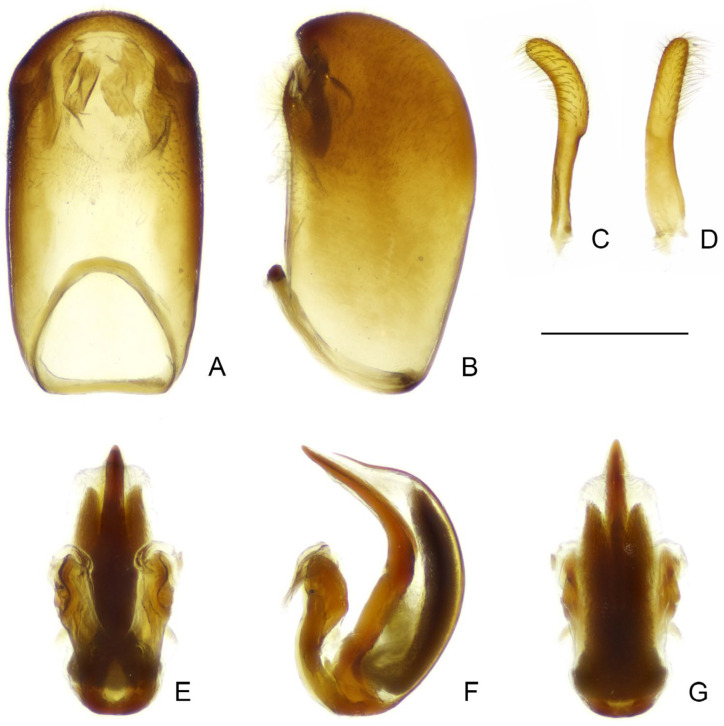
*Pleias trimaculata*  **sp. n.**, male genitalia. (**A**,**B**) pygophore; (**C**,**D**) paramere; (**E**–**G**) phallus. (**A**,**E**) Dorsal view; (**B**,**F**) lateral view; (**G**) ventral view. Scale bar of (**A**,**B**,**E**–**G**) = 1.0 mm; of (**C**,**D**) = 0.65 mm.

***Pleias trimaculata*** **sp. n.**

(Figure 16, Figure 17 and Figure 18)

*ZooBank LSID*: urn:lsid:zoobank.org:act:97BF2BED-3E99-409A-A3DF-E652E81FB1A8

**Type material. Holotype** (♂): “INDONESIA: \ SULAWESI UTARA, \ Dumoga-Bone N.P. \ July 1985.” [pr.]; “Fog 15 \ 400m 19.vii.85 \ BMNH Plot C” [pr.]; “TRAY \ 92” [pr.]; “♂” [pr.]; “HOLOTYPE [pr.] \ Pleias [hw.] \ trimaculata sp. n. [hw.] \ Des. Chen, Li & Cai [pr.]” [red rectangle] (BMNH). **Paratype**: “INDONESIA: \ SULAWESI UTARA, \ Dumoga-Bone N.P. \ July 1985.” [pr.]; “Fog 15 \ 400m 19.vii.85 \ BMNH Plot C” [pr.]; “TRAY \ 95” [pr.]; “♂” [pr.]; “PARATYPE” [pr., yellow rectangle] (1♂, BMNH).

**Diagnosis.** Medium-sized (11.7–12.1 mm) species that can be distinguished from its other congeners by the following character states: body generally yellowish brown (Figure 16); anterior lobe of pronotum 1.4 times as long as posterior lobe, largely dark brown except midline, lateral margins and dorsal margin of fore acetabulum (Figure 17A,B); posterior lobe of pronotum with three longitudinal, fuzzy-edged, dark-brown spots (Figure 17A,B); fore femur dark-brown on dorsal half and yellowish-brown on ventral half (Figure 17D); mid and hind femora blackish-brown at apices (Figure 16); fore wing with four whitish-yellow, vein-like stripes between R and M in apical half, cu-an1 meeting discal cell almost same level of second r-m (Figure 17E); pygophore with simple posterior margin (Figure 18A,B); phallus with elongate, sword-like, apically upturned dorsal sclerotization, and very narrow, band-like ventral sclerotization (Figure 18E–G).

**Description.** Macropterous male (Figure 16). ***Coloration.*** Generally yellowish brown (Figure 16). Head with dorsal surface brown, portion anteriad of antennifers and posterior margin darker. Antennal scape brown basally, gradually darkened towards apex, dark brown apically; pedicel and flagellomeres dark brown. Labium with visible segments I and II dark brown. Pronotum with contrasting dark and light color patterns; anterior lobe largely dark brown on each side (Figure 17A,B); posterior lobe with three longitudinal, dark brown spots with fuzzy edges, posterior margin slightly darkened, ventrolateral portion dark brown (Figure 17A,B). Episternal lobe largely blackish brown, epimeral lobe largely dark brown (Figure 17B). Scutellum and metanotum dark brown; meso- and metapleura blackish brown. Fore coxa with apical half (except extreme apex) of dorsal surface and ventral surface (except extreme base and apex) dark brown, with dark parts vaguely connected on outer surface subapically (Figure 17D); fore trochanter largely brown; fore femur blackish brown basally and apically, brown to dark brown on dorsal half of outer surface, with one brown patch at middle of inner surface (Figure 17A,B); fore tibia light brown, with base and apex slightly darker (Figure 17A,B); fore tarsus light brown (Figure 17A,B). Mid and hind femora brown basally, gradually darkened towards apex, blackish brown apically (Figure 16A,C); mid and hind tibiae yellowish brown basally, brown subbasally, gradually lightened towards apex, yellowish brown apically (Figure 16A,C). Forewing brown to dark brown; veins whitish yellow to yellowish brown (Figure 17E); area basad of discal cell with vague, whitish-yellow suffusion; apical half with four distinct, whitish-yellow, vein-like stripes between R and M (Figure 17E). Abdominal connexivum bicolorous, with each segment yellowish brown on basal half and dark brown on apical half; pygophore dark brown on apical half of lateral surfaces.

***Vestiture.*** As in redescription of the genus.

**Figure 19:**
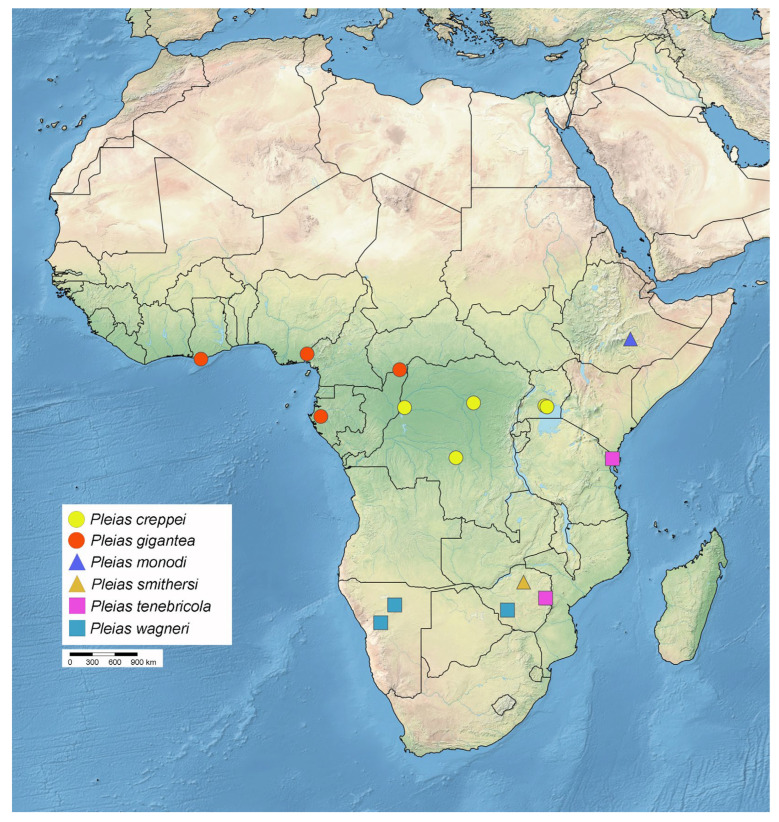
Known distribution of *Pleias* spp. in Africa.

**Figure 20:**
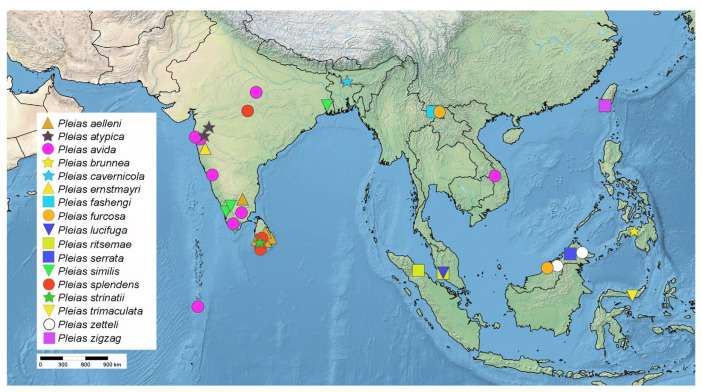
Known distribution of *Pleias* spp. in Asia.

***Structure.*** Head (Figure 17A–C) 1.15 times as long as width across eyes, two times as broad across eyes as interocular space; anteocular region 1.65 times as long as postocular; lateral margins of postocular region rounded in dorsal view; interocular furrow bisinuate. Pronotum (Figure 17A–C) 1.5 times as long as width across humeral angles; anterior lobe 1.4 times as long as posterior lobe, with shallow but distinct, median, longitudinal furrow; humeral angles rounded, weakly elevated in lateral view. Scutellum with apical margin nearly straight. Fore femur (Figure 17D) 1.65 times as long as fore coxa, 5.4 times as long as its maximum width; anteroventral series composed of about 43–44 spine-like setae arising from indistinct tubercles; posteroventral series composed of about 56 spine-like setae arising from indistinct tubercles; accessory series composed of small setigerous tubercles on basal fourth and small peg-like denticles on apical three-fourths of segment; fore tibia (Figure 17D) with about 28–34 deflexed spine-like processes ventrally; fore tarsus (Figure 17D) 0.7 times as long as fore tibia. Hind femur distinctly shorter than body length (to apex of abdomen). Forewing (Figure 17E) with cu-an1 meeting discal cell almost same level of second r-m; apical section of M distinctly shorter than discal cell. Abdomen 3.8 times as long as its maximum width; segment VIII with anteromedial and posteromedial margins nearly straight.

Male genitalia: Pygophore (Figure 18A,B) elongate oval, with rounded posterior margin; transverse bridge wide. Paramere (Figure 18C,D) thin, short, slightly curved, blunt at apex. Phallus (Figure 18E–G) robust, moderately sclerotized; articulatory apparatus thick, basal plate arms separated, basal foramen small and oval, ponticulus basilaris wide, dorsal connectives relatively long; basal plate extension short; phallosoma with elongate, sword-like, dorsal sclerotization curved upwards in apical half, and very narrow, band-like, ventral sclerotization slightly shorter than dorsal sclerotization; endosoma symmetrical, with complex arrangements inside.

**Measurements** [in mm, ♂ (n = 2)]. Length of body: to apex of forewings 11.90–12.30, to apex of abdomen 11.70–12.10; length of head 1.30; length of anteocular region 0.50; length of postocular region 0.30; width across eyes 1.10–1.15; interocular space 0.55–0.60; length of antennal segments I–IV = 8.30–8.50, 7.20–7.40, 1.70, 2.00; length of visible labial segments I–III = 0.40, 0.40, 0.50; length of pronotum 2.30; length of anterior pronotal lobe 1.35; length of posterior pronotal lobe 0.95; width of anterior pronotal lobe 1.00–1.10; width of posterior pronotal lobe 1.50–1.55; median length of scutellum 0.40; basal width of scutellum 1.00; length of fore coxa, femur, tibia, tarsus = 1.90–2.00, 3.20–3.30, 1.80–1.90, 1.30; maximum width of fore femur 0.60; length of mid femur, tibia, tarsus = 8.10–8.40, 11.10–11.30, 0.30; length of hind femur, tibia, tarsus = 10.60–10.90, 15.80–16.30, 0.30; length of fore wing 8.20–8.40; length of abdomen 6.40–6.50; maximum width of abdomen 1.70.

**Etymology**. The specific epithet is derived from Latin *tri-* and *-maculata*, referring to the three dark-colored markings on the posterior lobe of the pronotum.

**Distribution.** INDONESIA–Sulawesi: Bogani Nani Wartabone National Park.

**Comparative notes.** This is the first species of *Pleias* reported east of the Wallace Line. It can be easily separated from its other Asian congeners by the characteristic color patterns on the pronotum, legs, and forewing. The structures of the male genitalia make it similar to a group of medium-sized species from Southeast Asia, including *P. furcosa* **comb. n.**, *P. lucifuga* **comb. n.**, *P. serrata* **sp. n.**, *P. zetteli* **comb. n.**, and *P. zigzag*. This new species markedly differs from these species in the following respects: the pygohpore is nearly parallel-sided in dorsal view, with a wide transverse bridge and a simple posterior margin; the phallotheca has an elongate, sword-like dorsal sclerotization, which is curved upwards in the apical half, and a very narrow, band-like, ventral sclerotization, which is slightly shorter than the dorsal sclerotization.

### 3.4. Key to African Species of Pleias

1 Body length 18 mm or more; posterior lobe of pronotum with contrasting dark and light color patterns (Figure 2A,B and Figure 3C)…………………………………………………2

- Body length 13 mm or less; posterior lobe of pronotum uniformly brownish, lacking conspicuous color patterns (Figure 4A, Figure 5E, and Figure 6A,C,E)…………………………………3

2 Anterior lobe of pronotum 1.15 times as long as posterior lobe (Figure 2); posterior lobe of pronotum yellowish brown, with one pair of lobe-like, dark-brown patches on disc (Figure 2A,B)……………………………………………………………..***P. creppei*** **comb. n.**

- Anterior lobe of pronotum 1.5 times as long as posterior lobe (Figure 3E,F); posterior lobe of pronotum blackish brown, with wide, yellowish-brown, median band (Figure 3E)……………………………………………………………………….***P. gigantea*** **comb. n.**

3 Body length about 9 mm; anterior lobe of pronotum slightly longer than posterior lobe (Figure 4A,B); pygophore with short, flat, apically truncated median process………***P. monodi*** **comb. n.**

- Body length 10 mm or more; anterior lobe of pronotum distinctly longer than posterior lobe (Figure 6E,F); pygophore with triangular, apically rounded median process……4

4 Visible labial segment I not reaching anterior margin of eye (Figure 5F and Figure 6B); forewing with Cu delimiting basal margin of discal cell curved inwards to cell (Figure 5E and Figure 6B)…………………………………………………………………………………………5

- Visible labial segment I reaching anterior margin of eye (Figure 6F); forewing with Cu delimiting basal margin of discal cell straight (Figure 6C,E)……….***P. wagneri*** **comb. n.**

5 Forewing with cu-an1 meeting discal cell posterior to level of second r-m (Figure 6B); median process of pygophore wide………………………………***P. tenebricola*** **comb. n.**

- Forewing with cu-an1 meeting discal cell anterior to level of second r-m (Figure 5E); median process of pygophore narrow………………………………***P. smithersi*** **comb. n.**

### 3.5. Key to Asian Species of Pleias

1 Mid and hind femorotibial articulations uniformly brownish (Figure 13), or apices of femora blackish brown and bases of tibiae yellowish brown (Figure 16), but never whitish………………………………………………………………………………………………2

- At least hind femorotibial articulation whitish (e.g., Figure 6E,F, Figure 8A, and Figure 9A)………4

2 Posterior lobe of pronotum yellowish brown, with three dark-brown stripes (Figure 17A); forewing with contrasting dark and light color patterns (Figure 17E)………………………………………………………………………………………………………………………………………………………………………***P. trimaculata*** **sp. n.**

- Posterior lobe of pronotum rather unicolorous (Figure 14A); forewing lacking contrasting dark and light color patterns (Figure 14E)……………………………………….3

3 Body length about 9.5 mm; fore femur unicolorous; forewing with cu-an1 meeting discal cell slightly posterior to level of second r-m, apical section of M and discal cell subequal in length…………………………………………………………..***P. brunnea*** **comb. n.**

- Body length about 12 mm; fore femur with incomplete, light-brown annulus subapically (Figure 14D); forewing with cu-an1 meeting discal cell anterior to level of second r-m, apical section of M shorter than discal cell (Figure 14E)…………..***P. serrata*** **sp. n.**

4 Micropterous form; humeral angles of pronotum forming blunt, erect tubercles……..***P. atypica*** **comb. n.**

- Macropterous form; humeral angles of pronotum weakly to prominently elevated, but not forming tubercles………………………………………………………………………...5

5 Anterior and posterior lobes of pronotum subequal in length (e.g., Figure 1C,D)…….6

- Anterior lobe of pronotum distinctly longer than posterior lobe (e.g., Figure 1A,B)….9

6 Pronotum, foreleg, and forewing almost unicolorous…………………………………….7

- Pronotum, foreleg, and forewing with contrasting dark and light color patterns……..8

7 Body length about 7 mm, generally yellowish brown (Figure 4C–F); forewing with M + Cu and cu-an1 forming characteristic arched vein (Figure 4C,E)………….***P. ritsemae***

- Body length about 12.5–13.5 mm, generally brown (Figure 1C,D); forewing with M and Cu bifurcated more proximally, cu-an1 meeting discal cell posterior to level of second r-m…………………………………………………………………………..***P. avida*** **comb. n.**

8 Humeral angles of pronotum prominently elevated (Figure 10B); fore femur orangish brown, with dark-colored annuli (Figure 10D); abdominal connexivum bicolorous; pygophore with long, spine-like median process (Figure 11A,B)………..***P. fashengi*** **sp. n.**

- Humeral angles of pronotum weakly elevated (Figure 6B,D); fore femur blackish brown, with basal fourth yellowish brown (Figure 6B); abdominal connexivum unicolorous; pygophore lacking median process…………………………***P. splendens*** **comb. n.**

9 Pronotum with color patterns as shown in Figure 6E, anterior lobe 1.85 times as long as posterior lobe (Figure 6E,F); only hind femorotibial articulation whitish (Figure 6E,F)…………………………………………………………………….***P. strinatii*** **comb. n.**

- Pronotum with color patterns different, anterior lobe 1.15–1.75 times as long as posterior lobe; both of mid and hind femorotibial articulations whitish (e.g., Figure 8A and Figure 9A)……………………………………………………………………………………………10

10 Body length 9–10.5 mm; fore femur uniformly dark brown (Figure 1A,B) ……………………………………………………………………………………………………………………………………………………………………………….***P. aelleni*** **comb. n.**

- Body length 12 mm or more; fore femur with whitish subapical annulus (e.g., Figure 3B)…………………………………………………………………………………………….11

11 Pronotum with light-colored marking occupying posterior portion of anterior lobe and anterior half of posterior lobe; fore tibia whitish except base and apex dark-colored……………………………………………………………………………………………………………………………………………………………………..***P. cavernicola*** **comb. n.**

- Pronotum with color patterns different; fore tibia uniformly dark-colored, if with whitish subbasal annulus, then less than half length of segment……………………………12

12 Forewing with apical section of M nearly as long as discal cell (Figure 5A); pygophore with simple posterior margin………………………………………………………………13

- Forewing with apical section of M distinctly shorter than discal cell (Figure 3E); pygophore with short flattened elevation on posterior margin …………………………..14

13 Whitish-yellow patch on pronotum distinctly smaller in female (Figure 5C,D) than in male (Figure 5A,B); fore femur with whitish subapical annulus incomplete (Figure 5B,D); fore tibia uniformly dark-colored (Figure 5B,D)………………***P. similis*** **comb. n.**

- Whitish-yellow patch on pronotum of same size in both sexes; fore femur with whitish subapical annulus complete; fore tibia with whitish subbasal annulus ……………………………………………............................................................................................................................................................................................***P. ernstmayri*** **comb. n.**

14 Pronotum with whitish-yellow patch with deep bisinuate anterior and posterior margins; phallus with two superolateral groups of strongly sclerotized spines on phallosoma, and club-like dorsal projection on endosoma……………………………***P. zigzag***

- Pronotum with color patterns different; phallus with sclerotized processes in various shapes, and lacking above-mentioned dorsal projection on endosoma………………15

15 Forewing with apical section of M less than half of length of discal cell (Figure 3E); phallus with one pair of apically pointed superolateral processes, endosoma asymmetrical………………………………………………………………………***P. lucifuga*** **comb. n.**

- Forewing with apical section of M slightly shorter than discal cell; phallus with sclerotized processes in different shapes, endosoma symmetrical……………………………16

16 Posterior lobe of pronotum and fore tibia uniformly brown (Figure 3A,B); pygophore with one pair of small dorsal denticles; phallus with one pair of apically rounded superolateral processes…………………………………………………….***P. furcosa*** **comb. n.**

- Posterior lobe of pronotum with faint whitish-yellow patch occupying anterior half (Figure 8A); fore tibia with whitish subbasal annulus (Figure 8A); pygophore lacking above-mentioned denticles; phallus with one pair of apically narrowed superolateral processes…………………………………………………………………..***P. zetteli*** **comb. n.**

## 4. Discussion

### 4.1. Systematic Relationships of Pleias

Wygodzinsky [1] thought that *Pleias* (as *Bagauda*) belongs to a genus-group consisting of *Bettyella* Wygodzinsky, 1966 (9 spp., Malagasy), *Millotina* Villiers, 1953 (3 spp., Malagasy), and *Paraluteva* Villiers, 1961 (9 spp., Afrotropical, as *Barrosia* Villiers, 1952), but no further discussion was carried out. The molecular phylogenetic analysis of the Emesinae by Standring et al. [2] indicated that *Pleias* (as *Bagauda*) forms an early branch in the tribe Leistarchini, while *Bettyella* and *Paraluteva* (as *Barrosia*) are far distant from it. The phylogenetic relationships of the Leistarchini have not yet been resolved on the basis of large-scale taxa sampling, thus the exact phylogenetic position of *Pleias* remains unknown. Even though the following points warrant further discussion in future studies:

*Pleias* is morphologically similar and probably closely related to two monotypic Afrotropical genera, *Bagaudella* Miller, 1952, and *Pseudobagauda* Wygodzinsky, 1966, as well as the Asian *Guithera*-*Lutevula* group (which contains 1 sp. in *Guithera* Distant, 1906, 1 sp. in *Lutevula* Breddin, 1909, and 4 spp. in *Proguithera* Wygodzinsky, 1966), with the latter five genera being considered as close relatives in Wygodzinsky [1]. These taxa share the following morphological character states: dorsal surface of head more or less convex, with shallow transverse interocular sulcus, not forming deep incision in lateral view; ventral surface of head flattened, without spiniferous processes; pronotum robust, with well-developed posterior lobe nearly completely covering mesonotum; posterior margin of prosternum broadly rounded; fore tarsus distinctly longer than half length of fore tibia, strongly sclerotized, and if segmented, tarsomere I much longer than remaining tarsomere(s); similar forewing venation; somewhat elongate pygophore. Additional characters may also include the conditions of the anteroventral and posteroventral series of the fore femur and the presence of the accessory series on the fore femur. However, the accessory series of the fore femur is unclear in *Bagaudella* because it was not indicated in the original description, and the fore femora of the holotype of *B. whitfieldi* Miller, 1952 (the type species of *Bagaudella*) are missing. Among these genera, *Bagaudella* and *Pseudobagauda* may be more closely related to *Pleias*, whereas the *Guithera*-*Lutevula* group is more distinctive, exhibiting the more narrowed anteocular region of the head, the paired transverse tubercles on the posterior pronotal lobe, the reduced number of spine-like setae on the fore femur, and the fused fore tarsomeres. Available phylogenetic analyses never include the five genera mentioned above, and their potential phylogenetic proximity to *Pleias* needs to be tested in future studies.

The Afrotropical genus *Lhostella* Villiers, 1948 (8 spp.) also requires attention because of its similarity to *Pleias* in the structure of the head, foreleg, and forewing venation, and a number of species of *Lhostella* were originally described in *Pleias* (as *Bagauda*). As noted by Wygodzinsky [1], *Lhostella* differs from *Pleias* in the strongly shortened posterior lobe of the pronotum, the irregular rows of spine-like setae on the fore femur, and the peculiarly shaped struts of the males. Whether these two genera are systematically related deserves further evaluation.

### 4.2. Distribution of Pleias

The revised *Pleias* contains 23 species with a disjunct distribution in the Old World, with six species occurring in sub-Saharan Africa and the other 17 species distributed in southeastern Asia (Figure 19 and Figure 20).

The Afrotropical species present two different habitat preferences. Two large-sized and brightly-coloured species, *P. creppei* **comb. n.** and *P. gigantea* **comb. n.**, occur in the Congo Basin and along the Gulf of Guinea, where are dominated by tropical rainforests. The other four brown-colored species are found in more arid areas and are all cave-living species.

The distribution area of *Pleias* in Asia extends from the Indian subcontinent, through the Indochinese Peninsula, eastwards to the Malay Archipelago. The Indian subcontinent hosts the highest diversity of species, with six out of the eight species recorded in this region being endemic; most of the distribution records are concentrated in the Western Ghats and Sri Lanka, where it is listed as one of the world’s biodiversity hotspots, and the fauna is considered to be highly correlated [80]. Current distribution records show that several *Pleias* species are sparsely distributed in different localities in the Indochinese Peninsula, the Malay Peninsula, Sumatra, and Borneo, but there is still a large area in this region that lacks records of *Pleias*, with its potential diversity to be discovered in further surveys in the future. The present study also adds two noteworthy distribution records for the genus: a male of *P. avidus* **comb. n.** was collected from the southern Maldives, far from the mainland of Asia, suggesting the probability of transoceanic dispersal of the species; the newly described *P. trimaculata* **sp. n.** was found in Sulawesi, as the first representative of the genus known east of the Wallace Line. These records are important for interpretations of the biogeographic history of Asian *Pleias*.

The molecular phylogenetic analyses of the Emesinae by Standring et al. [2], sampling representatives of *Pleias* from Africa and Asia, recovered a monophyletic *Pleias* and showed that the Afrotropical species formed early branches, while the Oriental species clustered as a clade that was deeply nested within the Afrotropical branches. Combined within the known distribution records of the genus, it can be surmised that *Pleias* may have originated from Africa, subsequently dispersed to Asia through the Indian subcontinent, where it further diverged, and then continued to disperse to the other parts of Asia. Many cases of disjunct distribution in Africa and Asia have been found in the Reduviidae, and previous studies have demonstrated the close relationship among the Afrotropical, Malagasy, and Oriental reduviid faunas, and transoceanic dispersal may play an important role in linking these regions [81,82]. The biogeographic background of the distribution of *Pleias* is unclear, pending a time-calibrated phylogenetic analysis with comprehensive sampling to be established.

### 4.3. Ecology of Pleias

Species of *Pleias* are known for their cave-dwelling habits, which have been well documented in the early 1900s [47,60] and repeatedly reported in the century since [1,6,17,27,79]. Rédei [18] speculated that the cave-living species are endemic to the corresponding caves, and such a lifestyle may have played a role in their isolation and speciation. Some of these cave-living species were found occurring simultaneously with members of the emesine genus *Myiophanes* Reuter, 1881 [1,26,60]. In addition to the cave-living species, several cases of specimens collected outside of caves have been recorded in the genus [30,60,63]. Meanwhile, some observations also included the feeding habits of the species in concern [30,60,63]. Given that the above biological information may be valuable for the phylogenetic and evolutionary studies of the genus and even of the Emesinae, we here summarize two of the most fundamental aspects of the ecology of *Pleias*: habitat and feeding habits (Table 1).

From the known distribution records, it appears that some cave-dwelling species are not strictly restricted to a specific cave. For instance, *Pleias aelleni* **comb. n.** was recently reported from a number of different localities in Sri Lanka and India, and it was found not only in caves but also in wall crevices, tree holes, and habitats outside caves [8]; *P. furcosa* **comb. n.**, originally found in a cave in Borneo, is recorded from China in the present study, and specimens from both localities have almost identical morphological characters; *P. wagneri* **comb. n.** has been found in different localities in Namibia and Zimbabwe, respectively; and the cavernicolous *P. zetteli* **comb. n.** is presently found in caves in Sabah and Sarawak of Malaysia, respectively. Further field investigations may reveal more similar cases.

Species of *Pleias* show a diversity of feeding habits, known to include dipteran and lepidopteran insects as well as spiders, but the diet of many species remains unknown (Table 1). Three species from India (*P. atypica* **comb. n.**, *P. avida* **comb. n.**, and *P. ernstmayri* **comb. n.**) have been documented to be closely associated with spiderwebs, but there is no clear evidence of nutritional relationships between the thread-legged bugs and the spiders.

Future studies are recommended to focus on the ecology of the cave-living species, to clarify their life history, feeding habits, and behavior, and to compare them to the free-living species. This will help to understand their adaptations to cave environments and, in conjunction with phylogenetic analyses to explore the role of cave-dwelling lifestyle in the speciation of the genus, which provide a basis for conservation and evolutionary studies of *Pleias*.

## Figures and Tables

**Table 1:** Habitat and feeding habits of *Pleias*.

Species	Habitat	Feeding Habits	References
*Pleias aelleni* **comb. n.**	In cave; tree trunk	Lepidoptera; spider	[6,8]
*Pleias atypica* **comb. n.**	In cave	Spider	[27]
*Pleias avida* **comb. n.**	In cave	Spider	[29]
*Pleias brunnea* **comb. n.**	Unknown	Unknown	[48]
*Pleias cavernicola* **comb. n.**	In cave	Lepidoptera; spider	[60]
*Pleias creppei* **comb. n.**	In cave; tree trunk	Diptera	[49,63]
*Pleias ernstmayri* **comb. n.**	In cave	Spider	[26]
*Pleias fashengi* **sp. n.**	Tree trunk	Diptera	present study
*Pleias furcosa* **comb. n.**	In cave	Unknown	[17], present study
*Pleias gigantea* **comb. n.**	Unknown	Unknown	[49]
*Pleias lucifuga* **comb. n.**	In cave	Lepidoptera	[69]
*Pleias monodi* **comb. n.**	In cave	Unknown	[70]
*Pleias ritsemae*	Tree trunk	Unknown	[71]
*Pleias serrata* **sp. n.**	Unknown	Unknown	present study
*Pleias similis* **comb. n.**	Unknown	Unknown	[1]
*Pleias smithersi* **comb. n.**	In cave	Unknown	[1]
*Pleias splendens* **comb. n.**	Dense vegetation	Unknown	[60]
*Pleias strinatii* **comb. n.**	In cave	Unknown	[6]
*Pleias tenebricola* **comb. n.**	In cave	Unknown	[47]
*Pleias trimaculata* **sp. n.**	Unknown	Unknown	present study
*Pleias wagneri* **comb. n.**	In cave	Unknown	[77]
*Pleias zetteli* **comb. n.**	In cave	Unknown	[18], present study
*Pleias zigzag*	Tree trunk	Spider	[38]

## Data Availability

The original contributions presented in the study are included in the article, further inquiries can be directed to the corresponding author.
